# Cooperative amyloid fibre binding and disassembly by the Hsp70 disaggregase

**DOI:** 10.15252/embj.2021110410

**Published:** 2022-06-13

**Authors:** Joseph George Beton, Jim Monistrol, Anne Wentink, Erin C Johnston, Anthony John Roberts, Bernd Gerhard Bukau, Bart W Hoogenboom, Helen R Saibil

**Affiliations:** ^1^ Biological Sciences Institute of Structural and Molecular Biology Birkbeck University of London London UK; ^2^ Center for Molecular Biology of Heidelberg University (ZMBH) and German Cancer Research Center (DKFZ) DKFZ‐ZMBH Alliance Heidelberg Germany; ^3^ London Centre for Nanotechnology University College London London UK; ^4^ Department of Physics & Astronomy University College London London UK; ^5^ Present address: Centre for Structural Systems Biology (CSSB) Heinrich Pette Institute Leibniz‐Institute for Experimental Virology Hamburg Germany; ^6^ Present address: Leiden Institute of Chemistry Leiden University Leiden The Netherlands

**Keywords:** atomic force microscopy, cryo‐electron tomography, disaggregation, molecular chaperones, Neuroscience, Translation & Protein Quality

## Abstract

Although amyloid fibres are highly stable protein aggregates, a specific combination of human Hsp70 system chaperones can disassemble them, including fibres formed of α‐synuclein, huntingtin, or Tau. Disaggregation requires the ATPase activity of the constitutively expressed Hsp70 family member, Hsc70, together with the J domain protein DNAJB1 and the nucleotide exchange factor Apg2. Clustering of Hsc70 on the fibrils appears to be necessary for disassembly. Here we use atomic force microscopy to show that segments of *in vitro* assembled α‐synuclein fibrils are first coated with chaperones and then undergo bursts of rapid, unidirectional disassembly. Cryo‐electron tomography and total internal reflection fluorescence microscopy reveal fibrils with regions of densely bound chaperones, preferentially at one end of the fibre. Sub‐stoichiometric amounts of Apg2 relative to Hsc70 dramatically increase recruitment of Hsc70 to the fibres, creating localised active zones that then undergo rapid disassembly at a rate of ~ 4 subunits per second. The observed unidirectional bursts of Hsc70 loading and unravelling may be explained by differences between the two ends of the polar fibre structure.

## Graphical abstract

Structural and biophysical analyses show that chaperone coating of α‐synuclein fibrils precedes bursts of rapid, unidirectional disassembly.

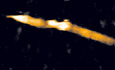

## Introduction

Neurodegenerative diseases are characterised by progressive aggregation of specific proteins, such as α‐synuclein (αSyn) in Parkinson’s disease, followed by neuronal cell death. Hereditary neurodegenerative diseases are caused by mutations that increase the cellular concentration or aggregation propensity of such proteins, suggesting a causal relationship between pathological aggregation and cell death. These pathological aggregates can form amorphous assemblies (Shahmoradian *et al*, 2019) and highly ordered cross‐β amyloid fibres (Fitzpatrick *et al*, 2017; Schweighauser *et al*, 2020). The identity of the toxic species remains unknown, with evidence for toxicity of smaller, disordered oligomers, thought to be precursors to amyloid fibres (Baglioni *et al*, 2006; Winner *et al*, 2011), and for toxicity of amyloid fibres (Peelaerts *et al*, 2015), which may accelerate disease by nucleating further oligomerisation (Padrick & Miranker, 2002; Guo *et al*, 2013). Given their strong association with disease, it seems likely that clearance of amyloid fibres is important to prevent disease onset (Kuo *et al*, 2013; Nagy *et al*, 2016).

Disaggregation in metazoans is mediated by Hsp70, which uses its ATP‐driven conformational cycle to dissolve aggregates (Mayer & Bukau, 2005; Gao *et al*, 2015; Nillegoda *et al*, 2015). Control of this Hsp70 ATPase cycle is provided by cognate J‐domain protein (JDP) and nucleotide exchange factor (NEF) co‐chaperones, which functionally tailor Hsp70s by controlling substrate selection and ATPase cycle progression. Indeed, a specific combination of the JDP DNAJB1, Hsc70 (the constitutively expressed human cytosolic Hsp70) and Apg2 (Hsp110 type NEF), is sufficient to disaggregate Tau, αSyn and huntingtin exon 1 amyloid fibres (Gao *et al*, 2015; Scior *et al*, 2018; Nachman *et al*, 2020). These amyloids are thought to be disassembled by Hsc70/DNAJB1/Apg2‐derived entropic pulling, caused by the entropic penalty that arises from steric clashes between chaperones bound to fibres at high density (Wentink *et al*, 2020).

DNAJB1 recruits Hsp70 to fibres and cannot be substituted by other JDPs in amyloid disaggregation (Gao *et al*, 2015; Wentink *et al*, 2020). This recruitment occurs through a two‐step mechanism initiated when the Hsp70 C‐terminal EEVD motif binds to DNAJB1. The initial binding releases the auto‐inhibition of DNAJB1’s J‐domain, which is then free to bind the Hsp70 ATPase domain, stimulating ATP hydrolysis (Faust *et al*, 2020). This two‐step binding process is necessary for DNAJB1‐facilitated clustering of Hsp70 on αSyn amyloid fibres, which is in turn necessary for disaggregation (Faust *et al*, 2020; Wentink *et al*, 2020). The force generated by the Hsc70/DNAJB1/Apg2 disaggregase is sufficient to fragment or depolymerise αSyn amyloid fibres, resulting in disaggregation (Gao *et al*, 2015). However, the dynamics of disaggregation are unknown. It remains unclear how the chaperones are organised at the fibre surface and how the amyloid fibres are remodelled during disaggregation, with data on these questions starting to emerge only recently (Franco *et al*, 2021; Schneider *et al*, 2021).

Here, we explore how the actions of the Hsc70/DNAJB1/Apg2 disaggregase lead to the disassembly of amyloid fibres, using αSyn fibres as a model substrate. We used atomic force microscopy (AFM) to visualise the disassembly process in real time. The resulting videos show how disaggregation proceeds by combined fragmentation and depolymerisation of individual fibres and reveal regions of long‐range chaperone clustering to be a necessary precursor for processive disassembly. Binding and activity assays, fluorescence microscopy and cryo‐electron microscopy (cryo‐EM) show that Apg2 recruits dense clusters of Hsc70 to the fibres, preferentially at one end, priming rapid bursts of disaggregation.

## Results

### Time‐resolved AFM of amyloid fibre disaggregation

**Figure 1:**
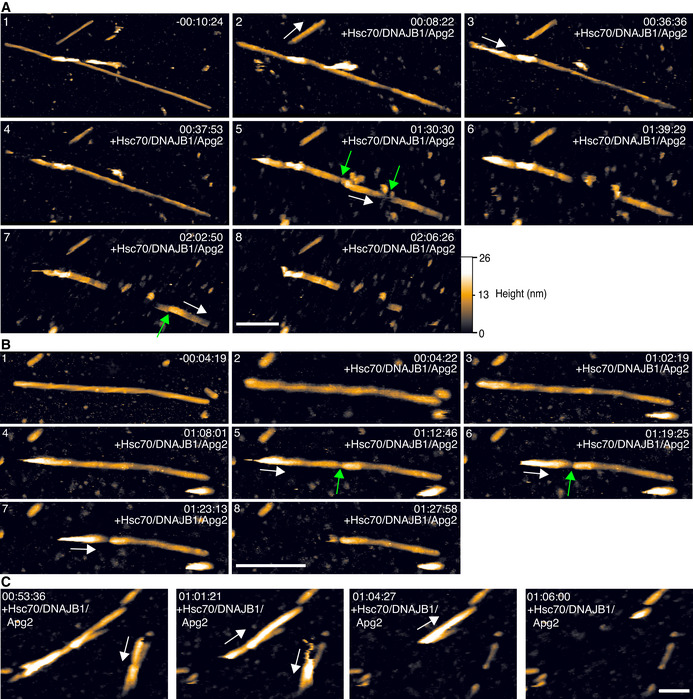
Frames from atomic force microscopy (AFM) movies of αSyn fibre disaggregation by Hsc70/DNAJB1/Apg2 An example video series showing the disaggregation of an αSyn fibre. The time stamp is in hours:minutes:seconds, with the chaperone/ATP mixture added at time 0:00:00. Scale bar, 250 nm. The height (colour) scale is 0 (black) to 26 nm (white), see inset at bottom right, where 0 nm refers to the passivated mica substrate.Video frames showing key stages of the disaggregation process. Time stamps and scale bar as in (A).Frames from AFM movie series that show the polar disassembly of αSyn fibres. The white arrows indicate the direction of depolymerisation. Time stamps as above and scale bar 200 nm. The videos are provided as expanded view movies EV1 and EV2. Data information: Green arrows indicate fragmentation sites. White arrows indicate the direction of depolymerisation along fibres.

To image the disaggregation process by AFM, we developed a system to adsorb αSyn fibres on a mica surface while avoiding excessive adsorption of the Hsc70/DNAJB1/Apg2 chaperones. We achieved this by incubating mica surfaces first with αSyn fibres and then with a PLL‐PEG copolymer. This PLL‐PEG has been shown to reduce non‐specific protein binding to mica surfaces in AFM experiments (Akpinar *et al*, 2019): the PLL component binds the negatively charged mica surface whilst the PEG component acts as a steric barrier to protein binding. Using this preparation, we could visualise αSyn fibres at the sample surface and greatly reduced non‐specific adsorption of subsequently added chaperones onto the PEG‐PLL passivated substrate. This enabled us to monitor the specific effect of added Hsc70/DNAJB1/Apg2 and ATP on αSyn fibres, including their resulting disaggregation. This disaggregation was visualised in eight video series in eight independent AFM experiments (Fig 1A and B; Movies EV1 and EV2), with a temporal resolution of 1–3 min per frame. This temporal resolution was suitable for stably imaging the surfaces over the longer time scales required to observe disaggregation events, yet was still fast enough to distinguish different steps in the disaggregation process.

The AFM movies EV1 and EV2 showed both fragmentation and runs of progressive depolymerisation (Fig 1B). This combined fragmentation and depolymerisation is consistent with published biochemical evidence (Gao *et al*, 2015). Not all disaggregating fibres were fragmented. Some were disassembled by multiple runs of unidirectional depolymerisation, and others appeared unaffected by the Hsc70/DNAJB1/Apg2 machinery. Of the 22 αSyn fibres imaged in eight videos, five fibres were not visibly affected by the Hsc70/DNAJB1/Apg2 disaggregase. There were no structural motifs evident by AFM that distinguished these intact fibres from those that were disassembled. Even for fibres that were disaggregated, disassembly was often incomplete (8 of 17 fibres were partially disaggregated; Fig 1A and B; Table EV1). The partial disaggregation was consistent with biochemical experiments which showed disaggregation can be incomplete (Gao *et al*, 2015; Wentink *et al*, 2020). For all observed cases of αSyn disaggregation, depolymerisation progressed in only one direction along a fibre (Fig 1C). The direction of depolymerisation was independent of AFM scanning direction, which excludes the AFM tip‐sample interaction as the cause of directional depolymerisation. Instead, this directionality indicates that depolymerisation is aligned with respect to the filament structural polarity. The polar nature of αSyn fibres has been demonstrated in multiple high‐resolution cryo EM reconstructions (Guerrero‐Ferreira *et al*, 2018, 2019; Li *et al*, 2018a; Li *et al*, 2018b), and has led to the hypothesis of a polar growth mechanism. Our AFM movies demonstrate that depolymerisation is polar.

In 6 out of the 8 of the movies, there was a delay of 30–60 min before any disassembly of αSyn fibres was observed (Fig 1A and B panels 2–3). In the other two movies of disaggregation, disassembly started almost immediately (5–10 min) after chaperones were injected into solution. Of note, no such delay was observed in biochemical assays of αSyn disaggregation (Gao *et al*, 2015; Wentink *et al*, 2020), implying that the disaggregation kinetics in AFM experiments may be slower due to the adhesion of the fibres to the solid support (mica). Alternatively, or additionally, fluid mixing may be less thorough in the AFM fluid cell than in solution, and may result in a slower build up of critical chaperone concentration at the fibre surface in the AFM experiment. Yet, once initiated, the chaperone‐mediated disaggregation was unambiguous (Fig 1).

**Figure EV1:**
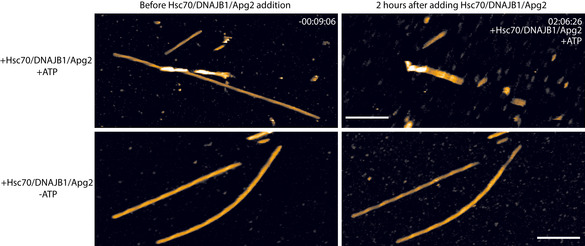
Disaggregation seen in AFM movies requires ATP Snapshots of an AFM time‐series showing αSyn fibres before addition of chaperones (left panels) and after incubation with chaperones for 2 h (right panels) in the presence (top panels) and absence (bottom panels) of ATP. The top panels show the same fibre as in Fig 1A and Movie EV1. The scale bars represent 250 nm.

To ensure that observed changes in fibre structure were due to bona‐fide chaperone activity, rather than due to interactions between the AFM tip and the sample, we tested the ATP‐dependency of αSyn fibril disaggregation in AFM videos. Hsc70/DNAJB1/Apg2‐mediated disaggregation requires ATP (Gao *et al*, 2015; Scior *et al*, 2018), whereas we expect mechanical perturbation by the AFM tip to be independent of ATP. In the absence of ATP, there was no visible disruption of αSyn fibres in the four AFM videos of fibres incubated with Hsc70/DNAJB1/Apg2 (Fig EV1). Therefore, the breaks and depolymerisation of αSyn fibres can be attributed to the action of the Hsc70/DNAJB1/Apg2 disaggregase, rather than to tip‐sample interactions related to the AFM imaging process.

### Amyloid fibres are depolymerised in rapid processive bursts

**Figure 2:**
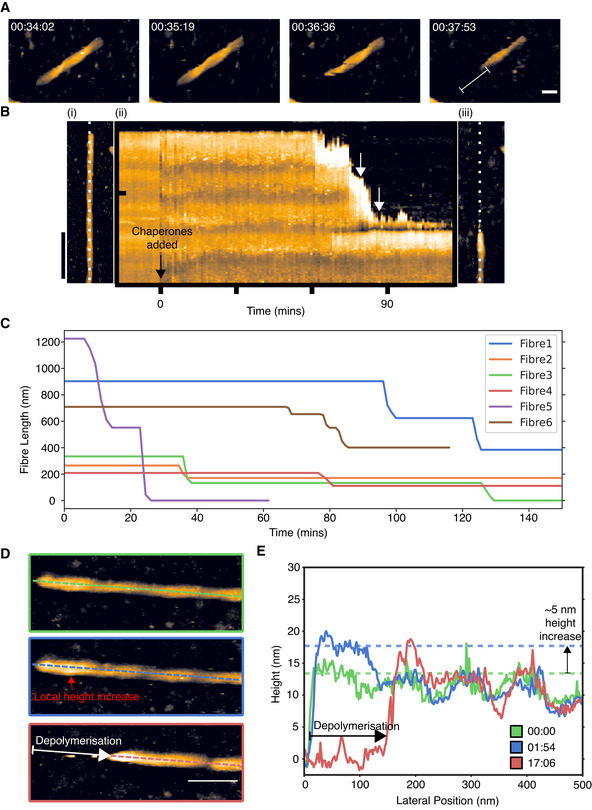
Time course of depolymerisation and morphological changes Example segment of an αSyn fibre depolymerised in a rapid “burst”, which removed 111 nm of this fibre (indicated with white marker) within two to three successive video frames (i.e. within 3–4 min). Height (colour) scale 26 nm, as in Fig 1. Scale bar: 50 nm.A kymograph showing the disaggregation of a single αSyn fibre: (i) The first frame of an atomic force microscopy (AFM) video series of disaggregation. Scale bar: 50 nm. (ii) Kymograph showing a height profile that is aligned to the fibre shown in A (white dotted line) as a function of time (horizontal axis of kymograph). Zero minutes is defined as the first frame recorded after the chaperones were injected into the solution. The white arrows indicate depolymerisation events. (iii) The same, now largely disaggregated, αSyn fibre as shown in A, at the end of the movie.Lengths of individual fibres plotted as a function of time through disaggregation reactions as imaged by AFM. Traces are shown for a subset of six fibres, each in a different colour, from four independent experiments.Disaggregation videos that show that a local height increase of αSyn fibres precedes depolymerisation. Zero time here refers to the latest frame before changes to the fibre were observed. Height (colour) scale: 21 nm, see Fig 1. Scale bar: 50 nm.Height profiles along the dashed lines in D showing the changes to αSyn fibres during disaggregation. The green trace was taken immediately before the increase in fibre height, and shows the undulations in fibre height due to its helical structure. The blue trace was recorded approximately 2 min after the change in fibre height (green trace), and it shows this height increase at the left‐hand end of the fibre. After about 17 min from the initial height change, the red trace shows the removal of the fibre over the same region that was previously elevated.

The AFM movies of αSyn fibre disaggregation revealed that depolymerisation occurs in short, rapid bursts. In these video series, segments of fibres were typically disassembled within two to three successive video frames, where each frame lasted 1–2.5 min (Fig 2A). The lengths of these rapidly depolymerised segments varied between 85 and 375 nm with an average of 176 ± 84 nm (mean ± SD, for 26 depolymerisation events from 17 fibres; initial and final fibril lengths are listed in Table EV1). We did not observe evidence of more gradual, continuous depolymerisation in any of the AFM movies. A total of 5 fragmentation events were observed over all AFM experiments (Fig 1).

**Figure EV2:**
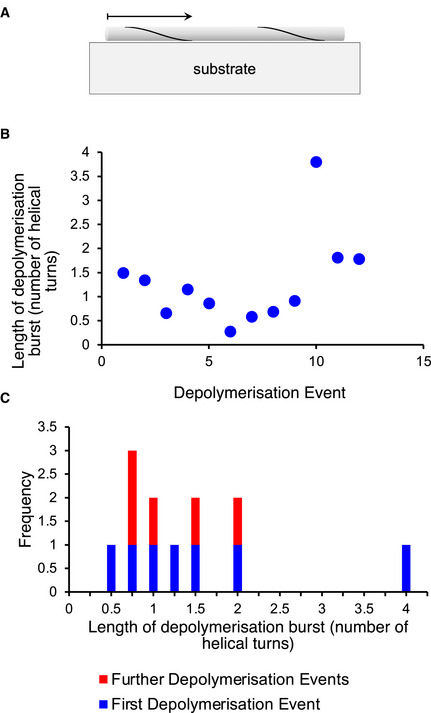
The helical periodicity does not account for the bursts of disassembly The chaperone binding sites are on the flexible αSyn termini, which are expected to follow the helical path of the fibre structure.We examined whether local attachment to the substrate might arrest disaggregation and account for the burst‐like depolymerisation events observed by AFM. No such relationship is evident between the helical repeat and the positions where disaggregation is arrested.The length of the first disaggregated segment is not less on average than that of subsequently disaggregated segments.

We considered whether the burst‐like nature of the depolymerisation reaction could be attributed to an artefact due to adhesion of the fibre to the mica substrate. Firstly, the chaperone binding and hence the depolymerisation reaction are expected to follow the helical path of the fibre structure (Fig EV2A). This helical fibre structure is detected in the AFM experiments prior to chaperone injection (Fig 1A and B) and is also visible as a height alternation in the AFM images (Fig 2D and E). Since a helical track at the fibre surface will periodically pass through the underside of the fibre, that is, the part of the fibre surface facing/adjacent to the solid mica support, we would expect a correlation between the lengths of the depolymerisation bursts and the helical periodicity if surface adhesion played a role in arresting depolymerisation. If the surface prevented progression of depolymerisation, the burst lengths should be limited by the helical periodicity and we would expect the disaggregated segment to be limited to lengths between 0 and one helical repeat, since the end of the attached fibre can be assumed to be at a random distance within one helical repeat of the point of the helix that is most firmly attached to the substrate. To test this, we plotted the burst lengths as a function of helical repeat in Fig EV2B, and compared the number of helical turns depolymerised for first and subsequent bursts in Fig EV2C. Since no correlation is observed between the helical repeat and the distribution of burst lengths, we rule out such an surface‐adhesion artefact (Fig EV2B). Finally, a specific prediction in the case of helically periodic adhesion would be that the first disaggregated segment would be on average ~ 50% of the length of subsequently disaggregated segments. The data in Fig EV2C show that this is not consistent with the experimental findings, providing further evidence against a surface‐adhesion artefact.

To assess the kinetics of depolymerisation, we translated the AFM movies into kymographs that displayed the height profile along a fibre as a function of time, with depolymerisation marked by a drop in measured height of the fibre thickness (8–12 nm) to the passivated mica substrate (~ 0 nm). These kymographs showed no evidence of gradual depolymerisation and further highlighted that depolymerisation occurred in stepwise bursts (Fig 2B). We quantified this by measuring lengths of individual fibres as a function of time, through the disaggregation movies (Fig 2C). Again, this highlighted that depolymerisation occurred exclusively by discrete stepwise bursts, as shown by sharp reductions in fibre length as a function time, and that fibre lengths were stable between such bursts (Fig 2C). These rapid depolymerisation events reduced the length of a fibre at an average speed of 64 ± 36 nm/min (mean± standard deviation; median value ~ 50 nm/min), whereas there were no observable changes in fibre length during the times (10–45 min) between such depolymerisation bursts (Fig 2B). The temporal resolution of the AFM videos was relatively low, typically two to three frames per depolymerisation burst. Nevertheless, it was sufficient for us to estimate the average depolymerisation speed during active bursts, corresponding to the extraction of ~ 4 monomers of αSyn from a fibre per second (for a 2 protofilament fibre). This stepwise depolymerisation, with bursts of depolymerisation interspersed with static dwells, indicates that the Hsc70/DNAJB1/Apg2 machinery is not able to continuously extract αSyn monomers from one end of a fibre to the other. We therefore hypothesised that αSyn fibres need to be locally destabilised before the Hsc70/DNAJB1/Apg2 machinery can proceed with αSyn removal.

### An increase in fibre height precedes depolymerisation

Consistent with the notion of a local change preceding depolymerisation, we noticed that αSyn fibre segments displayed a characteristic morphological change preceding their disassembly. Specifically, fibre segments underwent a uniform height increase of 4.2 ± 2.37 nm over lengths of short, 178 ± 65 nm segments (Fig 2D), correlating well with the 176 ± 84 nm length of disassembled segments as quantified for the disaggregation bursts as reported above. After this increase in height, there was a lag‐time of 2–5 min before the raised segment was depolymerised in a rapid burst, as described above. After depolymerisation, a similar morphological change (a short segment with increased height) was observed at the newly formed blunt end of the fibre (Fig 2D and E). In contrast to these depolymerisation events, we did not observe a similar morphological change prior to fibre fragmentation. Instead, breaks in fibres appeared spontaneously with no visible changes observed to precede their formation. However, we did see a height increase in the downstream (relative to the direction of depolymerisation) segment of fibres immediately after fragmentation, which was usually followed by depolymerisation.

**Figure EV3:**
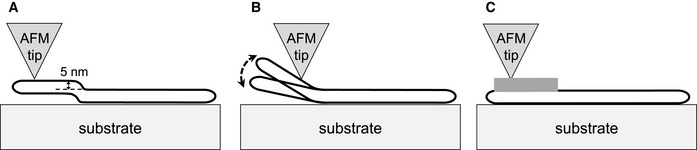
Alternative explanations of the fibre height increase seen by AFM A–CThe small and uniform height increase preceding a depolymerisation burst does not resemble local detachment from the mica. Local detachment from the mica would not result in a uniform height increase as in A, but would cause increasing mobility of the segment with distance from the detachment point, and a corresponding loss of imaging resolution (B). Dense chaperone binding over the elevated region (C) remains the only plausible explanation accounting for the AFM observations.

There are several potential explanations for the observed ~ 4 nm change in fibre height. Firstly, one might attribute the height increase to a partial detachment of the fibres from the surface before depolymerisation (Fig EV3A). Such detachment would cause a gradient of increasing freedom of movement of the fibre from the detachment point (Fig EV3B), rather than a small and uniform height increase (Fig EV3A). Moreover, AFM imaging of the detached region would have substantially reduced resolution due to fibre mobility. However, the experimental data show no such additional movement or loss of resolution. Alternatively, the increased height of the fibres might be caused by a change in fibre structure. However, previous negative‐stain EM tomograms did not reveal structural changes that would be consistent with a uniform increase in fibre height (Gao *et al*, 2015). Instead, we therefore attribute the local height increase to a local recruitment of Hsc70/DNAJB1/Apg2 to fibres (Fig EV3C), forming large and extended clusters that are necessary precursors for fibre disassembly.

### Cryo‐EM tomography of αSyn fibres with Hsc70/DNAJB1/Apg2 shows flexible but densely packed clusters of bound chaperones

**Figure 3:**
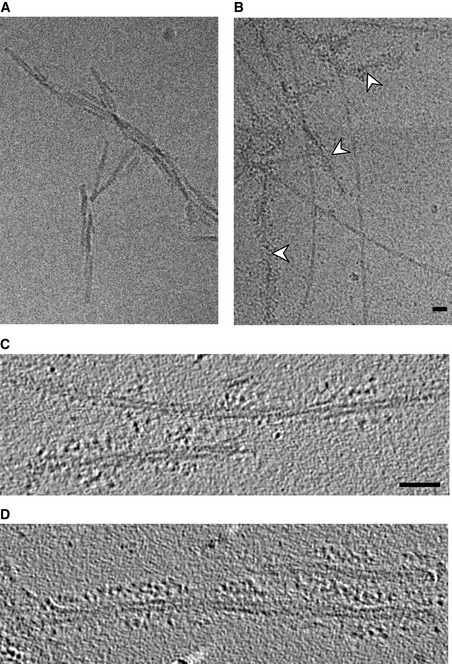
Cryo EM images and tomograms of fibre–chaperone complexes A, BRepresentative cryo EM images of αSyn fibres alone (A), αSyn fibres incubated with Hsc70/DNAJB1/Apg2/ATP (B). White arrowheads show sites of decoration.CTomogram section of fibres incubated with Hsc70, DNAJB1 and ATP. Small clusters are interspersed with sparse decoration.DTomogram section of fibres incubated with Hsc70, DNAJB1, Apg2 and ATP, showing extended stretches of densely clustered chaperones. The two‐protofilament structure of the fibres is evident, but the flexible αSyn N‐ and C‐terminal tails that contain the chaperone binding sites are not discernible. Data information: Scale bars, 30 nm.

To investigate the structural basis of the characteristic height increase preceding disaggregation, we visualised Hsc70/DNAJB1/ATP and Hsc70/DNAJB1/Apg2/ATP complexes on αSyn fibres by cryo‐EM. Cryo‐EM images of the fibres alone (Fig 3A) show ~ 10 nm‐thick helical filaments consistent with published data (Vilar *et al*, 2008; Guerrero‐Ferreira *et al*, 2018, 2019) and with the bare fibre height we observed by AFM. In cryo‐EM images of fibres in the presence of DNAJB1, Hsc70, Apg2 and ATP we observe chaperone clusters scattered along the fibres (Fig 3B).

Electron tomography of the decorated fibres shows clusters of elongated complexes, flexibly extending from the fibre surface, either in patches or more continuous arrays (Fig 3C and D). In the presence of Apg2, there are more extended regions with densely clustered chaperones (Fig 3D). These regions of continuous binding follow a roughly helical track, as expected from the underlying helical twist of the fibres (Movies EV3 and EV4).

The images and tomograms show that the chaperones extend away from the fibres, qualitatively consistent with the height increase in the AFM images. For such long and flexible complexes, we expect the AFM measurement to show reduced dimensions because of molecular compression and/or motion due to the force exerted by the AFM tip, which explains the difference between the 10–20 nm extension in the EM data and the ~ 4 nm height increase measured in the AFM experiments. In brief, the cryo‐EM and AFM data are consistent in showing local, dense recruitment of chaperones, which is required for localised bursts of depolymerisation.

### Hsc70 recruitment to the fibres requires both DNAJB1 and Apg2

**Figure 4:**
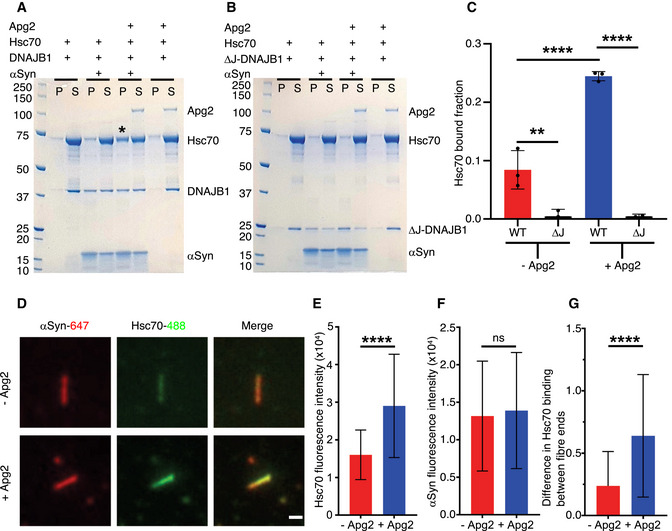
Hsc70 recruitment is greatly stimulated by Apg2 ABinding assay for Hsc70 binding in the presence of WT DNAJB1: control of DNAJB1/Hsc70, αSyn fibres + DNAJB1/Hsc70, αSyn fibres + DNAJB1/Hsc70/Apg2, control of DNAJB1/Hsc70/Apg2. The ‘*’ highlights the condition where an increase of Hsc70 in the bound fraction is observed. P, pellet; S, supernatant. The amount of Hsc70 recruited to the fibres is shown in the pellet fractions.BBinding assay for Hsc70 binding in the presence of the truncated ΔJ‐DNAJB1, lacking the J domain. The gel layout is similar to the previous one with ΔJ‐DNAJB1 replacing WT DNAJB1. Both Apg2 and J domain are required for enhancement of Hsc70 binding.CHistogram of Hsc70 bound fraction in each sample (*N* = 3 independent experiments, WT: WT DNAJB1, ΔJ: ΔJ‐DNAJB1 mutant, ‐Apg2: condition with fibres + DNAJB1/Hsc70, +Apg2: condition with fibres + DNAJB1/Hsc70/Apg2). A 2‐way ANOVA was performed with Tukey’s multiple comparisons test (**P = 0.003; ****P < 0.0001). Mean Hsc70 binding values with SD are shown.DTotal internal reflection fluorescence microscopy images of the labelled αSyn fibres and Hsc70 in the absence or in the presence of Apg2. Scale bar, 1 µm.E, FQuantitation of fluorescence intensity of Hsc70 (E) and αSyn (F) in the two conditions (+/− Apg2, *N* = 3 independent experiments, *n* = 65 fibres). Mean Hsc70 and αSyn fluorescence intensities with SD are shown.GQuantitation of differences in Hsc70 binding between the two ends of the fibres, normalised to the local αSyn intensity, in the absence and presence of Apg2 (*N* = 3 independent experiments, *n* = 54 fibres). Mean Hsc70 fluorescence intensities with SD are shown. Data information: (E–G) For each plot, data were tested for normality by performing a Shapiro–Wilk test. As the data did not display a normal distribution, they were analysed using a two‐tailed Mann–Whitney test (****P < 0.0001, ns: not significant).

**Figure EV4:**
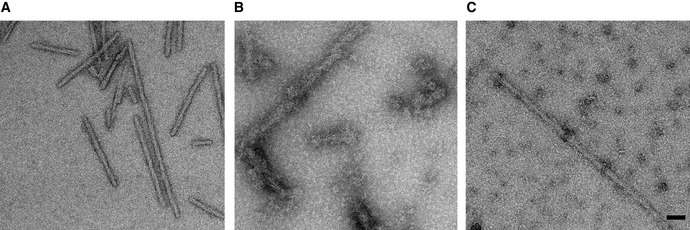
Negative stain EM images showing that chaperone recruitment to fibres did not occur when fibres were incubated with Hsc70/ΔJ‐DNAJB1/Apg2/ATP A–C In negative stain images, chaperone binding appeared as a pronounced increase in fibre thickness, visible when comparing images of αSyn samples alone to those incubated with Hsc70/DNAJB1/Apg2. (A) αSyn fibres alone; (B) fibres + Hsc70/DNAJB1/Apg2/ATP; (C) fibres + Hsc70/ΔJ‐DNAJB1/Apg2/ATP. Scale bar, 50 nm.

To determine the composition of the observed decoration, we performed biochemical assays quantifying chaperone binding to the fibres. Specifically, fibres were incubated with the chaperones and ATP, followed by separation into pellet (fibre) and supernatant (unbound) fractions. Comparing the binding with and without Apg2, we found a marked increase in recruitment of Hsc70 in the presence of Apg2 (Fig 4A). Controls in the absence of fibres were also carried out to confirm that Apg2 did not trigger the aggregation of Hsc70. In addition, the DNAJB1 dependence of Hsc70 binding to fibres was verified by comparison with a mutant version lacking the J domain which is primary site of contact between Hsc70 and J‐domain proteins (Fig 4B and C). Without the J domain, there was no Hsc70 recruitment detected by the binding assay in any of the conditions. In accordance with this lack of recruitment, negative stain EM of fibres incubated with ΔJ‐DNAJB1, Hsc70, Apg2 and ATP do not show decoration (Fig EV4). Fig EV4A shows fibres alone, EV4B shows fibres with Hsc70, DNAJB1, Apg2 and ATP, and Fig EV4C shows fibres with Hsc70, ΔJ‐DNAJB1, Apg2 and ATP. With wild type DNAJB1, Apg2 stimulation of Hsc70 recruitment was confirmed by total internal reflection fluorescence (TIRF) microscopy. Hsc70 fluorescence intensity was higher in the presence of Apg2 (Fig 4D–F), whereas the αSyn fluorescence intensity was the same in both conditions (Fig 4F). In agreement with AFM observations, TIRF intensities showed that Apg2 recruits Hsc70 preferentially to one end of each fibre (Fig 4G). This was done by comparing the Hsc70 fluorescence intensity, relative to the αSyn intensity, at the two ends of each fibre. Therefore, the binding assays show that initial recruitment of Hsc70 to the fibres requires the J domain of DNAJB1, and binding assays and TIRF confirm that Apg2 stimulates additional recruitment. The EM and TIRF microscopy together establish that the additional Hsc70 is recruited into dense clusters, enriched at one end of the fibres.

**Figure 5:**
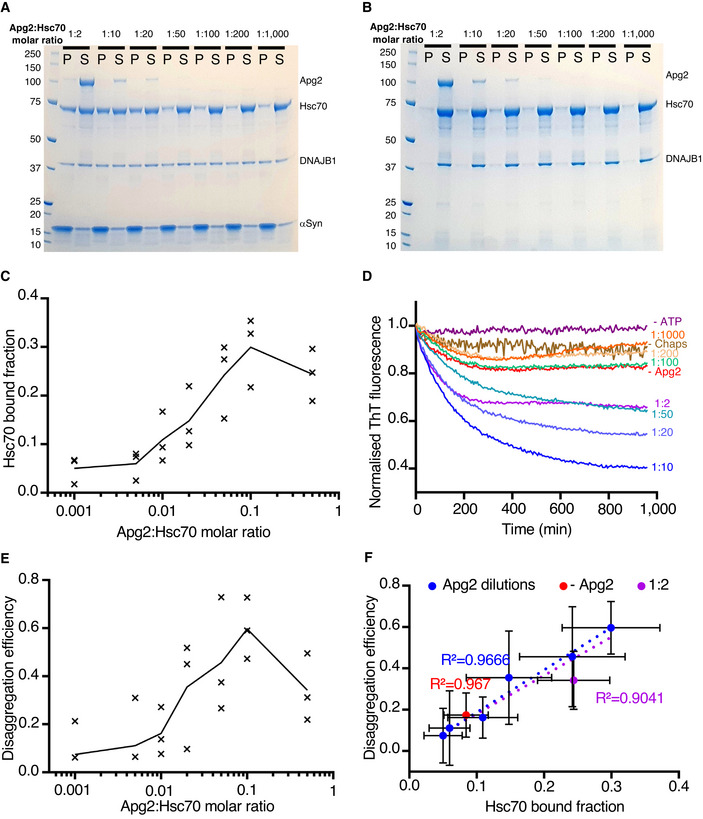
Dependence of Hsc70 recruitment and disaggregation activity on Apg2 concentration A, BBinding assay with different dilutions of Apg2 incubated with DNAJB1/Hsc70 and (A) with αSyn fibres or (B) without αSyn fibres (control). 5‐mM ATP was present in the buffer throughout.CPlot showing Hsc70 binding as a function of Apg2:Hsc70 molar ratio (*N* = 3 independent experiments). The action of Apg2 in recruiting Hsc70 to the fibres saturates at a molar ratio of Apg2:Hsc70 of 1:10. The curve indicates the mean Hsc70 binding values.DDisaggregation activity measured by Thioflavin T fluorescence shows the same dependence on Apg2 as binding assays. High Apg2 (1:2 molar ratio) makes disaggregation less efficient, possibly by prematurely releasing Hsc70.EQuantification of the disaggregation efficiency as a function of Apg2:Hsc70 molar ratio (*N* = 3 independent experiments). The disaggregation efficiency was determined by taking the values from the final hour of the assay curves in (D). The curve indicates the mean efficiency values.FPlot showing the linear correlation between disaggregation activity and Hsc70 binding. Including the data point without Apg2 did not change the correlation coefficient, which remained at 0.967. In contrast, including the highest Apg2 concentration (Apg2:Hsc70 molar ratio of 1:2) reduced the correlation coefficient to 0.904. Mean values with SD are shown.

We quantified the Hsc70 recruitment observed here as a function of Apg2 concentration. By performing a binding assay with different dilutions of Apg2, we found that substoichiometric Apg2 facilitates Hsc70 recruitment, saturating at a 1:10 molar ratio of Apg2:Hsc70 (Fig 5A–C). Apg2 concentration also affected the disaggregation efficiency (Fig 5D and E). Moreover, disaggregation activity shows the same dependence on Apg2 as binding assays (Fig 5F). This shows that a key function of Apg2 in disaggregation is its concentration‐dependent recruitment of Hsc70 into active assemblies.

The TIRF, binding and disaggregation assays establish that Hsc70 recruitment to the fibres is strongly stimulated by Apg2. This suggests that Apg2 is responsible for the increased chaperone clustering, even though it is a relatively minor component of the complex (At 1/10^th^ the concentration of Hsc70, it is not visible in the Coomassie‐stained pellet fractions, Fig 4). The effect of Apg2 saturated at a 1:10 molar ratio of Apg2:Hsc70 (Fig 5). There was no obvious change in DNAJB1 binding after Apg2 addition, despite the clear increase in Hsc70 binding. This does not preclude an essential role for DNAJB1: no Hsc70 binding was observed when the DNAJB1 was substituted by truncated DNAJB1 lacking the J‐domain and G/F linker (Fig 4B and C and negative stain images in Fig EV4). Our findings show that the large chaperone clusters seen on Hsc70/DNAJB1/Apg2‐incubated fibres are composed predominantly of densely packed Hsc70 and DNAJB1. The binding of DNAJB1 was previously estimated as one dimer every 5 nm (Gao *et al*, 2015), and the spacing of the globular features in Fig 3D is roughly 6 nm. This suggests that Hsc70 and DNAJB1 are similar in mass/unit length of fibre, within a factor of ~ 2.

## Discussion

In this study, we have used AFM to directly visualise disassembly of αSyn fibres by the Hsc70/Apg2/DNAJB1 disaggregase in real time. In agreement with previous work (Gao *et al*, 2015), the AFM movies show that Hsc70‐mediated disaggregation of αSyn fibres proceeds by combined fragmentation and depolymerisation. Additionally, the movies provide further insight into the disaggregation mechanism: αSyn fibres were disassembled by polar depolymerisation that occurred in rapid bursts, in which short fibre segments were disassembled within 2–5 min. These depolymerisation bursts are facilitated by stretches of densely clustered chaperones, as observed by cryo EM, and appearing as a local height increase along fibre segments in the AFM images just prior to disaggregation bursts. The clusters consist largely of Hsc70 and DNAJB1, but substoichiometric amounts of Apg2 are required to stimulate further Hsc70 recruitment and to create the extended, dense clusters. Although fragmentation events were less frequent than depolymerisation bursts, they could contribute to disaggregation by providing new sites for initiation of disaggregation bursts. In addition, they are a potential source of fibril seeds that can be trafficked between cells and could therefore be important in a disease context.

Two recent papers report related observations: Schneider *et al* (2021) measure diffusion coefficients on a microscopic scale to follow the release of monomers during disaggregation, and Franco *et al* (2021) image fibre disaggregation by high‐speed AFM to resolve protofilament unwinding at the ends of fibres undergoing rapid depolymerisation. The observed unwinding is very localised and transient, and the speed of fibre shortening is roughly 2–4 nm/s. Our estimate from lower time resolution data is about 1 nm/s, a good agreement given differences in experimental conditions such as chaperone concentrations and AFM substrate functionalisation and passivation. Neither of those studies reported fibril fragmentation events, and they did not follow the chaperone binding during disassembly.

Previous studies have shown that Hsc70 clustering is necessary for amyloid disaggregation, but the FRET analysis in those studies did not reveal Apg2‐stimulated additional recruitment of Hsc70 to the fibrils (Faust *et al*, 2020; Wentink *et al*, 2020). We have directly visualised these clusters by cryo electron tomography, showing that Hsc70/DNAJB1/Apg2 forms densely packed chaperone clusters that stretch over extended (>~ 100 nm) lengths along the αSyn fibres.

Earlier negative stain EM tomography showed that DNAJB1 binds all along the fibres, and suggested that Hsc70 and Apg2 individually bind irregularly to the fibres, although the additional densities were not identified biochemically (Gao *et al*, 2015). It has been shown that the JDP plays a crucial role in recruiting Hsc70 to the fibres, with a specific requirement for release of the auto‐inhibitory interaction in DNAJB1 (Faust *et al*, 2020). In addition, Hsc70 clustering driven by specific Apg2 NEF activity is important for disaggregation (Wentink *et al*, 2020). The FRET experiments of Wentink *et al* were not designed to look for increased Hsp70 loading, and the increase in recruitment was not detectable by fluorescence anisotropy because of the high background of unbound protein, a problem circumvented in our binding assays and TIRF experiments. Our new findings reveal that Apg2 increases the recruitment of Hsc70 (Fig 5), a role that was not previously reported. Our analysis of the complexes reveals an important, dose‐dependent effect of Apg2 in recruiting additional Hsc70 into dense clusters on the fibres, and in stimulating the bursts of disaggregation. It is surprising that a recycling factor (NEF), which is expected to release Hsc70 from its substrate, increases total Hsc70 bound to the fibrils. A possible explanation is that NEF stimulation increases Hsc70 recruitment even more than it increases release.

Our AFM movies suggest that chaperone clustering is a necessary and direct precursor to processive depolymerisation of the fibres, consistent with the previous demonstration that cluster formation is required for fibre disassembly. Interestingly, we observe such clusters predominantly at fibre ends in TIRF and AFM images. In combination with the observed unidirectional depolymerisation, this suggests that the Hsc70/DNAJB1/Apg2 machinery preferentially pre‐assembles at one end of the polar αSyn fibres (Guerrero‐Ferreira *et al*, 2018, 2019; Li *et al*, 2018a) for rapid, processive depolymerisation.

**Figure 6:**
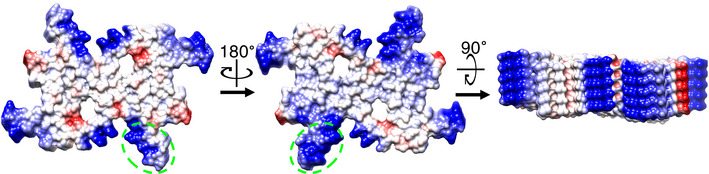
Polarity of the Hsc70 binding site in the ordered part of the fibre structure The polar structure of the fibre is shown with electrostatic surface colouring, showing a crucial Hsc70 binding site on αSyn with a more favourable exposure of positive charge (blue) on one surface than on the other. The Hsc70 binding site 37–43 is outlined in green on one protofilament. This figure shows PDB 6cu7 (B. Li *et al*, 2018; Data ref: B Li *et al*, 2018) which has a very similar fold to fibres used in much of our analysis.

Aiming to uncover a possible structural basis for polar disassembly, we considered the two preferred Hsc70 binding sites on αSyn, residues 1–10 and 37–43 (Wentink *et al*, 2020). Deletion of the disordered N terminus removes the 1–10 site, but Wentink *et al* showed that the resulting fibres can still be disaggregated, to a level about 60% of the full‐length protein. However, fibres lacking the 37–43 site are completely resistant to disaggregation by the Hsc70 system. Since this site is part of the cross‐β fold, we decided to examine it in the available αSyn fibre structures (Li *et al*, 2018a; Li *et al*, 2018b; Guerrero‐Ferreira *et al*, 2019). In all but one of the wild‐type fibre structures, there is a difference in the electrostatic surface exposed by this site at the two ends of the polar fibres. This is shown in Fig 6 for one of the fibre structures. Peptide binding by Hsp70 was shown to favour a mixture hydrophobic and positive side chains (Rüdiger *et al*, 1997). This polar exposure might favour Hsc70 interaction with the crucial 37–43 site at one end more than the other. Alternatively, or additionally, one end of the fibre may be less stable than the other because of the intrinsic structural polarity. This hypothesis is consistent with observations of polar assembly (faster fibre growth in one direction along a fibre than the other) of Aβ1‐42 fibres (Watanabe‐Nakayama *et al*, 2016; Young *et al*, 2017), the functional bacterial amyloid curli (Sleutel *et al*, 2017) and for subpopulations of the Sup35 yeast prion (DePace & Weissman, 2002).

**Figure 7:**
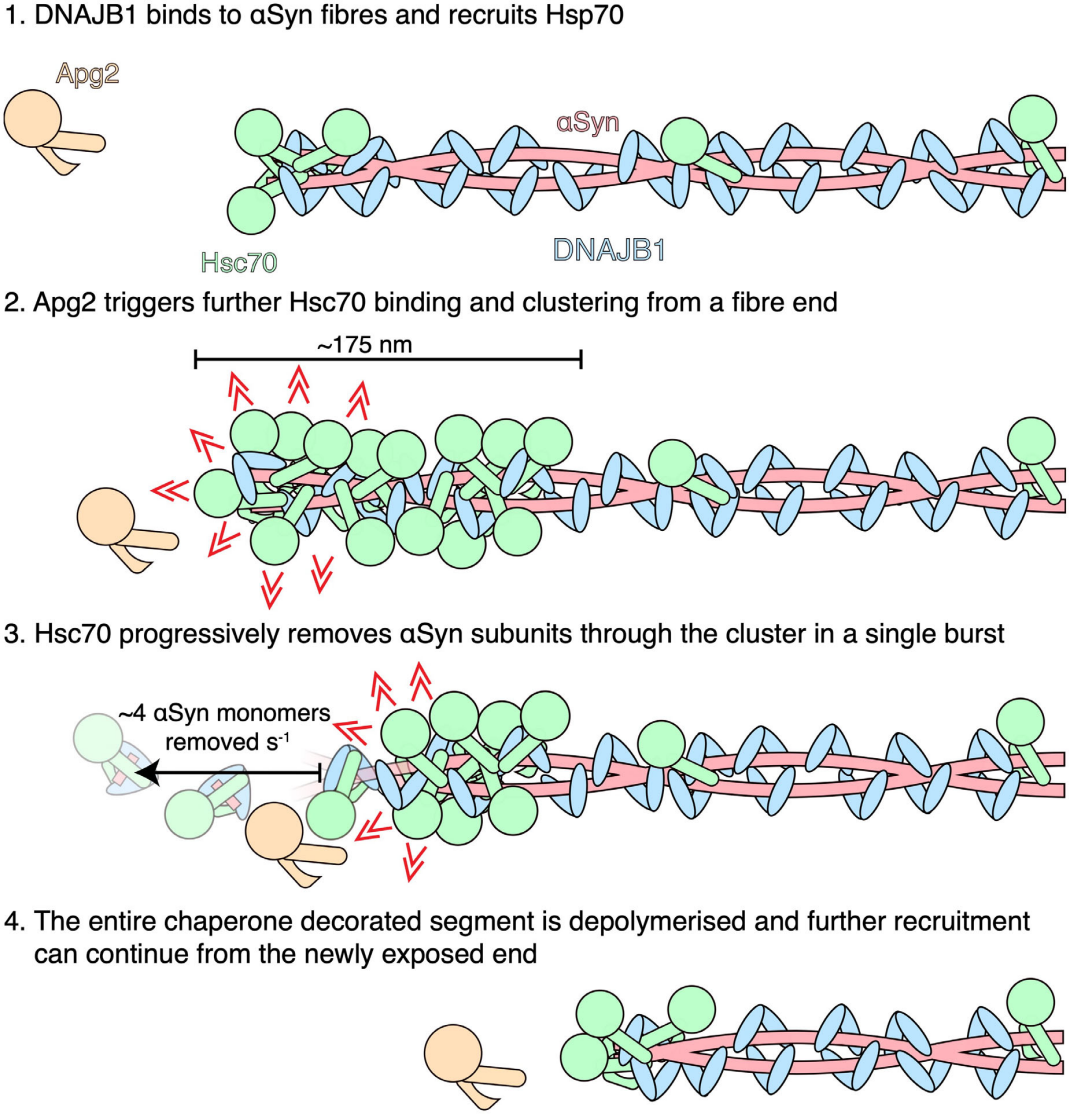
Proposed model of αSyn fibre depolymerisation Apg2 catalyses the assembly of densely packed clusters of Hsc70, priming the rapid bursts of disaggregation from one end of the fibre.

Based on these observations, we propose a model for αSyn fibre depolymerisation, initiated when Hsc70 binds to the 37–43 αSyn‐binding site which is more favourably exposed at one fibre end than the other (Fig 7). We speculate that this binding triggers the progressive recruitment of a dense cluster of Hsc70s on the fibre. Wentink *et al*, 2020 showed that dense clustering is required to generate sufficient pulling force to destabilise the fibre. We now propose that this occurs mainly from one end, priming the loaded fibre segment for subsequent rapid and cooperative extraction of αSyn monomers by the recycling Hsc70/DNAJB1/Apg2 complex. Once initiated, this depolymerisation then proceeds rapidly along the whole decorated segment.

We speculate that this depolymerisation could represent the reversal of amyloid assembly: one hypothesis for amyloid nucleation is that the process is initiated by oligomer formation followed by a conformational rearrangement that results in the amyloid structure (Chatani & Yamamoto, 2018; Nors Perdersen *et al*, 2015). The massive local recruitment of the Hsp70 disaggregase machinery could be required to provide sufficient force to overcome the activation energy for partial amyloid unfolding, forming a more disordered aggregate which can then be rapidly disassembled.

In summary, we have presented the first views of the dense arrays of chaperones that bind along segments of αSyn fibres to form tracks that subsequently trigger bursts of rapid, unidirectional disassembly. It remains a future challenge to understand the dynamics of the Hsc70 system during these bursts.

## Materials and Methods

### Hsc70 and Apg2 expression and purification

Hsc70 and Apg2 were expressed in *E. coli* Rosetta™ DE3 cells with an N‐terminal His_6_‐Smt3 tag and purified by nickel affinity chromatography. Protein expression was induced using IPTG. Cell lines were grown in LB media, at 37°C, to an OD_600_ density of 0.6–0.8 and 0.5 mM IPTG was added to the cell cultures. After induction, cell cultures were incubated for a further 3 h before cells were harvested by centrifugation (4,200 *g*, 20 min).

Hsc70 and Apg2 were purified by identical protocols. Pellets from respective cell lines were resuspended in Hsc70/Apg2 lysis buffer (Table EV2), with an EDTA free protease inhibitor tablet (Roche), and lysed by cell disruption. Cell debris and insoluble protein was removed by centrifugation (48,000 *g*, 60 min) before the soluble fraction was collected and incubated with Ni‐IDA beads for 2 h at 4°C. Contaminant proteins were removed from the column in three successive washes: first using Hsp70/110 wash 1 buffer, followed by Hsp70/110 wash 2 buffer, and finally 2 washes with ATP wash buffer (Table EV2). Hsc70 and Apg2 were isolated using on column cleavage in which the resin was incubated with Ulp1 protease at 4°C overnight. After this, the flow through was collected and the column was washed twice with Hsp70/110 lysis buffer and these fractions were collected. Hsc70 samples were concentrated to 85 µM and Apg2 samples were concentrated to 25 µM. Samples were aliquoted, flash frozen in liquid nitrogen and stored at −80°C. The T111C Hsc70 (T111C, C267A, C574A, C603A) mutant was expressed and purified as described above. DTT was replaced by TCEP in all the buffers.

### DNAJB1 expression and purification

DNAJB1 was expressed using the same construct design as for Hsc70 and Apg2 (with an N‐terminal His6‐Smt3 tag) and with the same protocols. DNAJB1 was purified using nickel affinity purification and size exclusion chromatography (SEC). Cell lines for DNAJB1 were lysed by cell disruption in DNAJ lysis buffer, the resulting sample was centrifuged (48,000 *g*, 60 min) and the soluble fraction was collected and used for purification.

For DNAJB1 purification, the construct was bound to an Ni‐IDA column as described above for Hsc70/Apg2 and contaminant proteins were removed from the column by two successive wash steps, using DNAJ wash‐1 buffer followed by DNAJ wash‐2 buffer (Table EV2). DNAJB1‐His6‐Smt3 was eluted with DNAJ elution buffer (Table EV2) and collected. The His6‐Smt3 tag was removed using Ulp1 during overnight dialysis into DNAJ lysis buffer (Table EV2). After the tag was cleaved, the solution containing DNAJB1 was reapplied to a Ni‐IDA column to remove Ulp1, the His6‐Smt3 tag and uncleaved DNAJB1‐His6‐Smt3. The flow through and two column washes with DNAJ lysis buffer fractions were collected and further purified by SEC.

SEC was performed using a Superdex 200 Increase 10/300 gel filtration column (GE Healthcare) using DNAJ SEC buffer (Table EV2). Pure fractions were collected and concentrated to ~250 µM. Samples aliquoted, flash frozen in liquid nitrogen and stored at −80°C.

The purified truncated DNAJB1 mutant lacking the J‐domain and the G/F linker (ΔJ‐DNAJB1) was kindly provided by Rina Rosenzweig (Weizmann Institute). Briefly, the mutant was purified by ion metal affinity chromatography (IMAC, Nickel), dialysed and cleaved with TEV protease, subjected to reverse IMAC and then subjected to SEC. The protein was stored in 25 mM HEPES pH 7.5, 200 mM KCl, 10 mM MgCl_2_, 2 mM DTT.

### αSyn purification

αSyn was expressed as a native untagged sequence in DE3 Star *E. coli* cell lines (Thermo Fisher Scientific, USA) by identical protocols to those described above for Hsc70/Apg2. Cell lines were lysed by cell disruption in αSyn lysis buffer (Table EV2), the resulting sample was centrifuged (48,000g, 60 min) and the soluble fraction was collected. This soluble fraction was boiled for 20 min to aggregate *E. coli* cellular proteins and centrifuged (21,000 *g*, 30 min) to pellet the aggregates. *E. coli* nucleic acids were precipitated by adding 30 mg/ml streptomycin sulphate to the soluble fraction which was then incubated for 30 min at 4°C. The resulting solution was centrifuged (20 min, 20,000 *g*) to pellet the precipitated nucleic acids and the supernatant was collected. αSyn was precipitated by adding 400 mg/ml ammonium sulphate and incubating the resulting mix at 4°C for 30 min. The resulting sample was centrifuged (20 min, 20,000 *g*), the supernatant was discarded, and the pellet, containing αSyn, was resuspended in TBS buffer (Table EV2) and dialysed overnight into deionised water.

The dialysed sample was purified by ion exchange chromatography with a HiTrap Q HP column using αSyn buffer A and B followed by further purification by SEC using αSyn SEC buffer (Table EV2). Pure fractions were collected and used to form amyloid fibres.

The S9C αSyn mutant was expressed and purified as described above. TCEP was added to a final concentration of 2 mM in the purification buffers.

### αSyn fibrillation reaction

We produced αSyn fibres as previously described (Gao *et al*, 2015): 1 ml of 200 µM monomeric αSyn was incubated in fibrillation buffer (Table EV2) in a 1.5 ml Protein LoBind Eppendorf tube (Eppendorf, Germany) on an orbital shaker (PCMT Thermoshaker Grant‐bio) at 1,000 rpm, 37°C for 1 week. We confirmed amyloid fibres had formed, rather than amorphous or small aggregates, by negative stain electron microscopy. S9C:WT (1:2, molar ratio) αSyn fibres were prepared as described above. TCEP was added to a final concentration of 2 mM in the fibrillation buffer.

### AFM sample preparation

αSyn fibres were immobilised on freshly cleaved mica disks (diameter 12 mm) by non‐specific adsorption: 20–50 μM αSyn fibres (monomer concentration) were added to a 20 μl droplet of HEPES buffer (Table EV2) on a mica surface. This sample was incubated at room temperature for 30 min after which the surface was washed 3 times with 50 μl HEPES buffer.

We used a poly(ethylene glycol)‐block‐poly(L)‐lysine (PEG‐PLL) (Alamanda Polymers, USA) copolymer composed of 22 repeats of polyethylene‐glycol (PEG) and 10 repeats of poly‐L‐lysine (PLL) to reduce non‐specific binding of chaperones to the mica surface (Akpinar *et al*, 2019). A layer of PEG‐PLL was added to the remaining mica surface after αSyn adsorption by adding 0.5 μl 1 mg/ml PEG‐PLL to the 20 μl HEPES buffer droplet. This was incubated for 1 min before the surface was thoroughly washed 10 times with 60 μl HKMD buffer (Table EV2).

In preparation for disaggregation reactions, the buffer was exchanged by three washes with 50 μl disaggregation buffer (Table EV2) or with HKMD buffer for controls without ATP. After buffer exchange, 40 μl disaggregation buffer (HKMD for ‐ATP controls) was added to the sample droplet. A 20‐μl droplet of disaggregation buffer (HKMD buffer for ‐ATP controls) was added to the cantilever loaded on to the Z‐scanner (FastScan) or fluid cell (Multimode 8). The final sample volume was ~80 μl.

### AFM imaging of disaggregation

All AFM imaging was performed in liquid using Peakforce Tapping with either a Dimension FastScan microscope (images presented in Figs 1A and B, and 2A and D, and EV1) or a Multimode 8 (images presented in Fig 1C). FastScan D cantilevers (Bruker) were used with the Dimension FastScan microscope and PeakForce HIRS‐F‐B cantilevers (Bruker) were used with the Multimode 8 microscope. FastScan D cantilevers were operated using a drive frequency of 8 kHz and PeakForce HIRS‐F‐B cantilevers were operated at 4 kHz drive frequency. The areas imaged were 0.8 × 0.25–4 × 2 µm^2^ in size and recorded at 3.5 Hz (FastScan) or 1.75 Hz (Multimode 8) line rate. Images were 512 × 256, 512 × 172 or 384 × 172 pixels in size.

During AFM movie acquisition, disaggregation was initiated by retracting the cantilever tip ~100 nm from the surface and injecting 0.75–1 µM Hsc70, 0.37 – 0.5 µM DNAJB1 and 0.07–0.1 µM Apg2 (always at a Hsc70:DNAJB1:Apg2 molar ratio of 1:0.5:0.1) in disaggregation buffer (HKMD buffer for ‐ATP controls) into the sample droplet. AFM imaging was continued within 1 min of injection of chaperones. The same area of αSyn fibres was imaged for >2 h at either at room temperature or at 30°C.

### AFM image processing

AFM images were processed using the Gwyddion 2.52 software package (Nečas & Klapetek, 2012). Raw data was initially corrected using plane‐subtraction to remove sample tilt. After this, images were corrected for systematic height errors between fast‐scan (*x*‐axis) line profiles using median background subtraction for each line. Additional distortions were removed by second‐order polynomial subtraction, using a polynomial calculated using only pixels corresponding to the mica/PEG‐PLL surface. Higher‐frequency noise was reduced by a Gaussian filter with a full width at half maximum of 2–3 pixels, corresponding to 3–8 nm. The height values in the image were offset such that the mean pixel value was set to zero. The colour scale was set to an appropriate range (listed in the Figure legend for each image) and images were saved in TIFF format.

Images corresponding to a video series were opened as an image sequence using the ImageJ Fiji software package (Schindelin *et al*, 2012). Images in each time‐series were aligned as rigid bodies using the Fiji module “StackReg” (Thévenaz *et al*, 1998) to correct for lateral drift between frames. Aligned movies were then cropped and saved as individual TIFF images and as AVI movies for analysis.

### Cryo EM sample preparation

αSyn amyloid fibres were sonicated for 30 min using a Branson 1800 ultrasonic cleaner to produce dispersed fragments of αSyn fibres. The suspension was diluted to 6 µM αSyn monomer concentration and mixed, when indicated, with 6 µM Hsc70, 3 µM DNAJB1 and 0.6 µM Apg2, and incubated for 1 h at 30°C in disaggregation buffer (or HKMD buffer for the control without chaperones). 4 μl of this preparation were applied to negatively glow discharged holey carbon C‐flat grids (CF‐1.2/1.3‐4C) (Protochips, USA), back blotted and plunge frozen in liquid ethane using a Leica EM GP2 (Leica Microsystems, Germany).

For the cryo‐electron tomography experiments, similar samples were prepared. The sample was applied to glow discharged holey carbon grids which were then back blotted before adding 3 μl of 10 nm Protein‐A‐coated gold (EMS, USA) as fiducial markers for 3D reconstruction of tilt series. The grids were then back blotted a second time before plunge freezing.

### Negative stain sample preparation

αSyn fibres (10 µM monomer) were mixed, when indicated, with Hsc70 (10 µM), Apg2 (1 µM), and WT or ΔJ‐DNAJB1(5 µM monomer) for 1 h at 30°C in the disaggregation buffer (or HKMD buffer for the control without chaperones). These samples were applied to glow discharged continuous carbon film‐coated, 300 mesh copper grids (EMS) and blotted after 1 min. The grid was then stained with 2% (w/v) uranyl formate and immediately blotted three times.

### EM data collection

Images displayed in Fig 3 and negative stain images in supplementary Fig 4 were acquired using a Tecnai TF20 electron microscope (Thermo Fisher, USA) equipped with FEG source operating at 200 keV. Images were recorded with a DE‐20 direct electron detector (Direct Electron, USA) at a magnification corresponding to 1.83 Å/pixel using a total dose of ~ 30 e^−^/Å².

Tilt series of samples without or with Apg2 were acquired on the Titan Krios at Birkbeck College at 300 keV using a BioQuantum energy filter and post GIF K3 detector (Gatan, USA). 10‐frame movies were acquired per tilt angle. Tilt series were acquired with SerialEM (Mastronarde, 2005) from −60° to +60° with a 3° increment using a dose symmetric tilt‐scheme with a super resolution pixel size of 1.065 Å and a 2–5 μm defocus range. The total dose was around 150 e^−^/ Å². 30 and 23 tilt series were collected of the conditions without or with Apg2 respectively. Frames underwent whole‐frame alignment in MotionCor2 version (Zheng *et al*, 2017) and were dose weighted before reconstructing the tomograms using IMOD version 4.9.0 (Kremer *et al*, 1996). Tomograms were binned by 4 in X and Y. The tomogram of the sample with Apg2 was CTF‐corrected in IMOD.

### Sedimentation assay

All chaperone aliquots (Hsc70, DNAJB1, Apg2) were centrifuged at 17,000 *g* for 30 min at 4°C. αSyn amyloid fibres were sonicated for 15 min at high frequency using a CPX 2800 Bransonic Ultrasonic bath (Branson). αSyn amyloid fibres (40 µM, monomer concentration) were incubated with Hsc70 (8 µM), DNAJB1 or ΔJ‐DNAJB1 (4 µM), and Apg2 (different concentrations as indicated) in the disaggregation buffer for 1 h at 30°C. The samples were then centrifuged for 45 min at 17,000 *g* to separate the pellet, containing the fibres and bound chaperones, from the supernatant with unbound chaperones. The supernatant was then collected and both fractions were incubated in 4X NuPAGE LDS Sample buffer (Thermo Fisher Scientific) for at least 30 min at 90°C. Samples were then loaded on BOLT 4–12% Bis‐Tris gels (Thermo Fisher Scientific) and proteins were separated by SDS‐PAGE. At least three independent experiments were performed for each condition. The statistical analyses were performed in Prism 8 (GraphPad). Data were analysed using a two‐way ANOVA test with Tukey’s multiple comparisons test.

### Thioflavin T assay

In order to keep the αSyn amyloid fibres well dispersed and accessible to the chaperones, they were sonicated for 15 min at high frequency using a CPX 2800 Bransonic Ultrasonic bath (Branson). Thioflavin T (ThT) fluorescence was recorded every 5 min for 16 h on a FLUOstar Omega plate‐reader (BMG LABTECH, excitation: 440 nm, emission: 482 nm) to monitor the disaggregation of αSyn amyloid fibres. αSyn amyloid fibres (2 µM, monomer concentration) were incubated with Hsc70 (4µM), DNAJB1 (2 µM), and Apg2 (different concentrations as indicated) in the disaggregation buffer and 15 µM ThT. Background ThT fluorescence of chaperones and buffer was subtracted and ThT fluorescence was normalised to the fluorescence intensity of the first time point (t = 0 min). At least three independent experiments were performed for each condition. The disaggregation efficiency was calculated by averaging the values of the final hour for each experiment. The plots of normalised intensity over time as well as the linear regression showing the disaggregation efficiency as a function of Hsc70 bound fraction were plotted and calculated in Microsoft Excel (for Windows 365, version 16.0.13127.21624).

### TIRF microscopy sample preparation

**Figure EV5:**
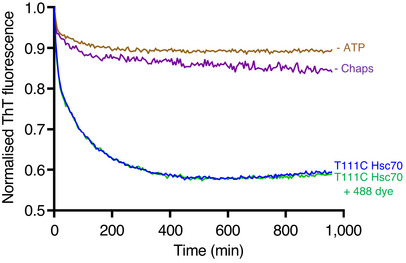
Disaggregation activity measured by Thioflavin T fluorescence Labelling T111C Hsc70 mutant with the 488 maleimide dye does not affect the efficiency of the disaggregation.

S9C:WT (1:2) αSyn amyloid fibres were incubated with Alexa Fluor 647 maleimide dye (10‐fold molar excess, Thermo Fisher Scientific) and Biotin‐X, SE dye (2.5‐fold molar excess, Thermo Fisher Scientific) for at least 2 h at room temperature in the dark in HKMT buffer. T111C Hsc70 was incubated with Alexa Fluor 488 maleimide dye (10‐fold molar excess, Thermo Fisher Scientific) for at least 2 h at room temperature in the dark in HKMT buffer. Labelled Hsc70 was then buffer exchanged using PD SpinTrap G‐25 column (Cytiva) pre‐equilibrated in HKMD buffer. Labelled amyloid fibres (1 µM) were incubated with labelled Hsc70 (2 µM), DNAJB1 (1 µM) and Apg2 (0.2 µM, if indicated) in disaggregation buffer for 1 h at 30°C in the dark. The T111C‐Alexa Fluor 488 Hsc70 had the same disaggregation activity as unlabelled T111C‐Hsc70 (Fig EV5).

### TIRF microscopy imaging and analysis

Chambers were prepared using glass slides, biotin‐PEG coverslips, and double‐sided tape. Chambers were passivated with 0.5% BSA for at least 10 min, washed twice in HKMD buffer, incubated twice in 0.5 mg/ml neutravidin for 2 min, washed three times in HKMD buffer, incubated with the sample for 10 min, and washed three times in HKMD buffer. Samples were imaged on an Eclipse Ti‐E inverted microscope with a CFI Apo TIRF 1.49 N.A. oil objective, Perfect Focus System, H‐TIRF module, LU‐N4 laser unit (Nikon) and a quad band filter set (Chroma), described in Toropova *et al*, 2017. Frames were recorded on an iXon DU888 Ultra EMCCD camera (Andor), controlled with NIS‐Elements AR Software (Nikon) with an exposure time of 100 ms.

To determine the overall fluorescence of the fibres, Hsc70 and αSyn fluorescence intensities were measured in ImageJ Fiji software package by determining the plot profile of 65 fibres for each condition. The minimum value for each plot profile was considered as background and subtracted. At least 3 independent experiments were performed for each condition. The statistical analysis were performed in Prism 8 (GraphPad). Data were first analysed by running a Shapiro–Wilk test to check their normality. A two‐tailed Mann Whitney test was then performed as the data did not have a normal distribution.

To evaluate the distribution of bound Hsc70, Hsc70 and αSyn fluorescence intensities were measured along the fibres. The intensities in the first and last 3 pixels of each fibre were compared: the ratio of Hsc70 intensity divided by αSyn intensity was calculated at each end and the lower ratio was then subtracted from the higher one. The division by αSyn intensities served to minimise the influence of variations in fibre distance from the glass surface. The difference between the two ratios was plotted in Fig 4G. A 54 fibres from three independent experiments were measured. Statistical analyses were performed as described above using Prism 8 (Shapiro–Wilk test followed by a two‐tailed Mann–Whitney test as the data did not have a normal distribution).

## Author contributions

**Joseph George Beton:** Investigation; Methodology; Writing—original draft; Writing—review & editing. **Jim Monistrol:** Investigation; Methodology; Writing—original draft; Writing—review & editing. **Anne Wentink:** Conceptualization; Investigation; Methodology; Writing—original draft; Writing—review & editing. **Erin C Johnston:** Investigation. **Anthony John Roberts:** Supervision; Funding acquisition; Methodology; Writing—original draft; Writing—review & editing. **Bernd Gerhard Bukau:** Conceptualization; Funding acquisition; Writing—original draft; Writing—review & editing. **Bart W Hoogenboom:** Conceptualization; Supervision; Funding acquisition; Methodology; Writing—original draft; Writing—review & editing. **Helen R Saibil:** Conceptualization; Supervision; Funding acquisition; Writing—original draft; Writing—review & editing.

In addition to the CRediT author contributions listed above, the contributions in detail are:

JB, JM, AW and ECJ performed experiments and analysed data. HRS, BB, AW, BWH, JB, JM and AJR designed experiments. All authors contributed to writing the manuscript.

## Disclosure and competing interests statement

HRS and BB are editorial advisory board/EMBO Members. This has no bearing on the editorial consideration of this article for publication.

## Supporting information



Expanded View Figures PDF

Movie EV1

Movie EV2

Movie EV3

Movie EV4

Table EV1

Table EV2

## Data Availability

Tomograms: EM databank: EMD‐13576 https://www.ebi.ac.uk/emdb/EMD‐13576 and EMD‐13577 https://www.ebi.ac.uk/emdb//EMD‐13577; TIRF data: BioImage archive: S‐BIAD415 https://www.ebi.ac.uk/biostudies/studies/S‐BIAD415; AFM images: University College London Research Data Repository: https://doi.org/10.5522/04/19524907; EM images: Birkbeck Research Data Archive: https://doi.org/10.18743/DATA.00191; SDS PAGE binding assays: Birkbeck Research Data Archive: https://doi.org/10.18743/DATA.00190.
