# Supplementary material for: Cooperative amyloid fibre binding and disassembly by the Hsp70 disaggregase
Source: EMBO J. 2022 Jun 13;41(16):e110410. doi: 10.15252/embj.2021110410 (PMC9379549; doi:10.15252/embj.2021110410)
Supplement: Supplementary file 1 — Expanded View Figures PDF [file EMBJ-41-e110410-s001.pdf]

Expanded View Figures

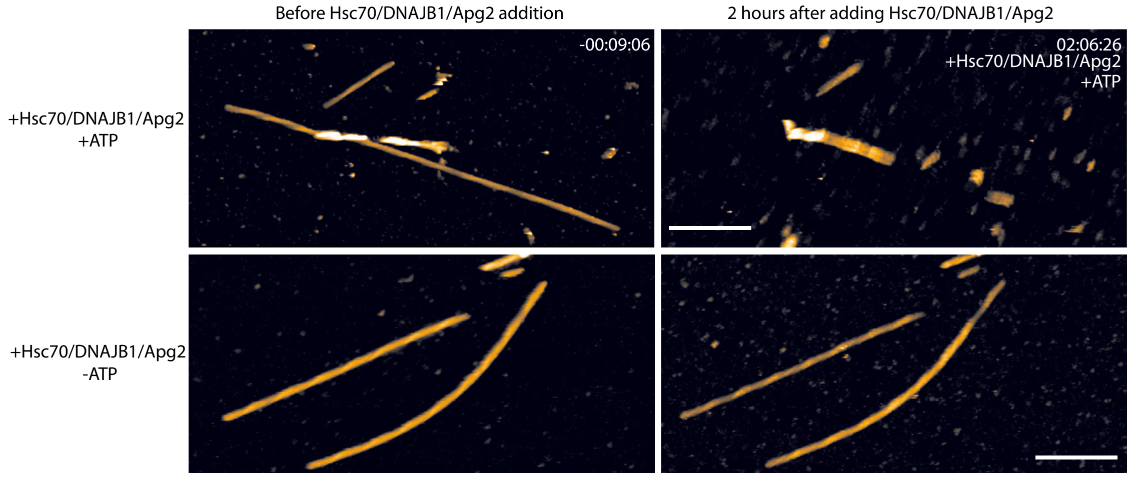

Figure EV1. Disaggregation seen in AFM movies requires ATP.

Snapshots of an AFM time-series showing αSyn fibres before addition of chaperones (left panels) and after incubation with chaperones for 2 h (right panels) in the presence (top panels) and absence (bottom panels) of ATP. The top panels show the same fibre as in Fig 1A and Movie EV1. The scale bars represent 250 nm.

© 2022 The Authors

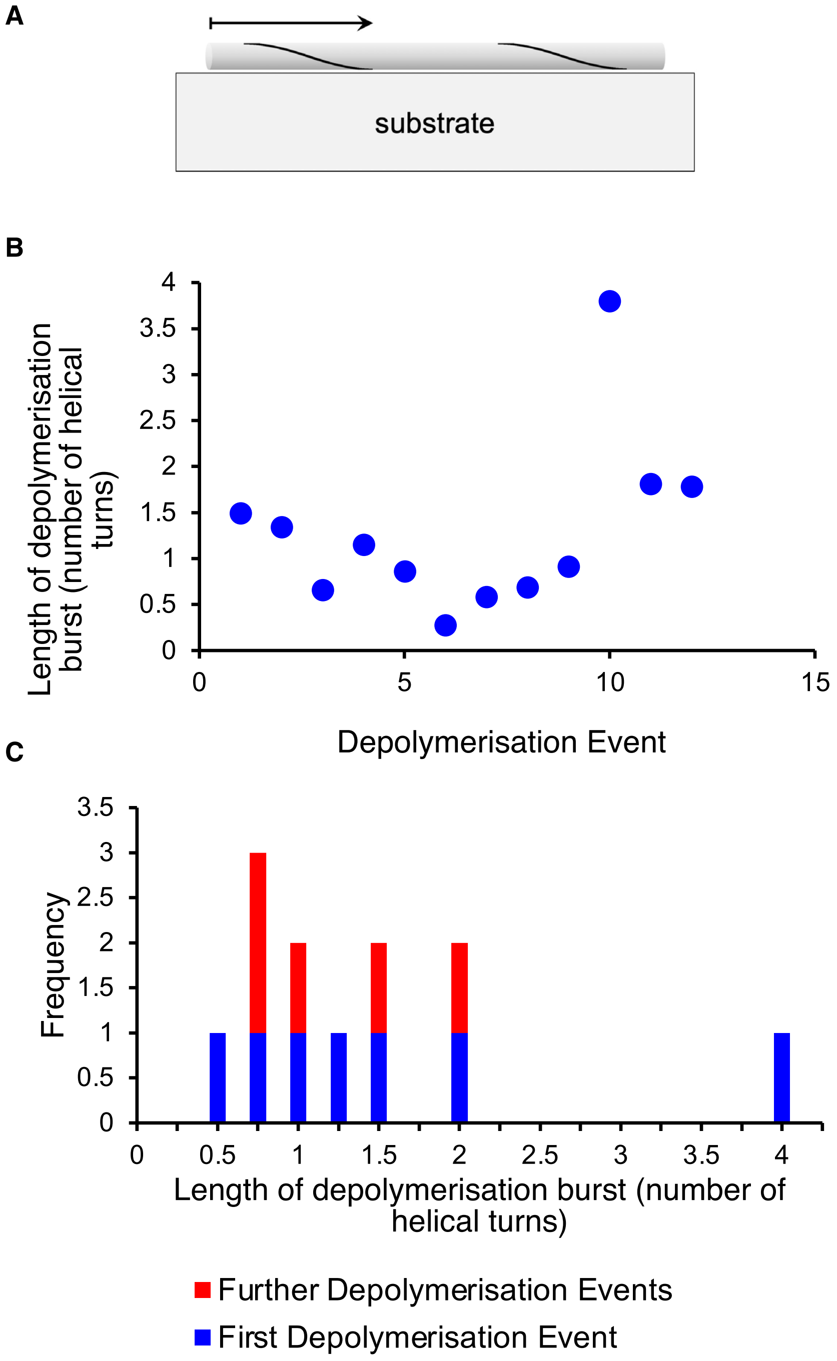

Figure EV2. The helical periodicity does not account for the bursts of disassembly.

A The chaperone binding sites are on the flexible αSyn termini, which are expected to follow the helical path of the fibre structure.

B We examined whether local attachment to the substrate might arrest disaggregation and account for the burst-like depolymerisation events observed by AFM. No such relationship is evident between the helical repeat and the positions where disaggregation is arrested.

C The length of the first disaggregated segment is not less on average than that of subsequently disaggregated segments.

© 2022 The Authors

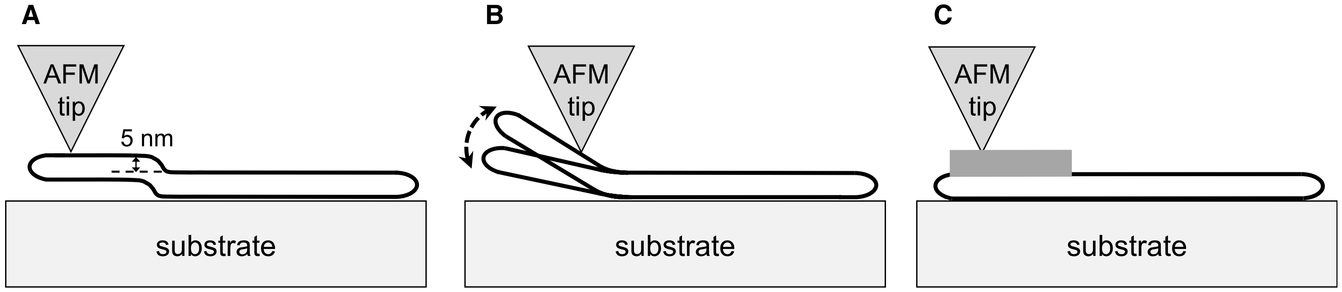

Figure EV3. Alternative explanations of the fibre height increase seen by AFM.

A–C The small and uniform height increase preceding a depolymerisation burst does not resemble local detachment from the mica. Local detachment from the mica would not result in a uniform height increase as in A, but would cause increasing mobility of the segment with distance from the detachment point, and a corresponding loss of imaging resolution (B). Dense chaperone binding over the elevated region (C) remains the only plausible explanation accounting for the AFM observations.

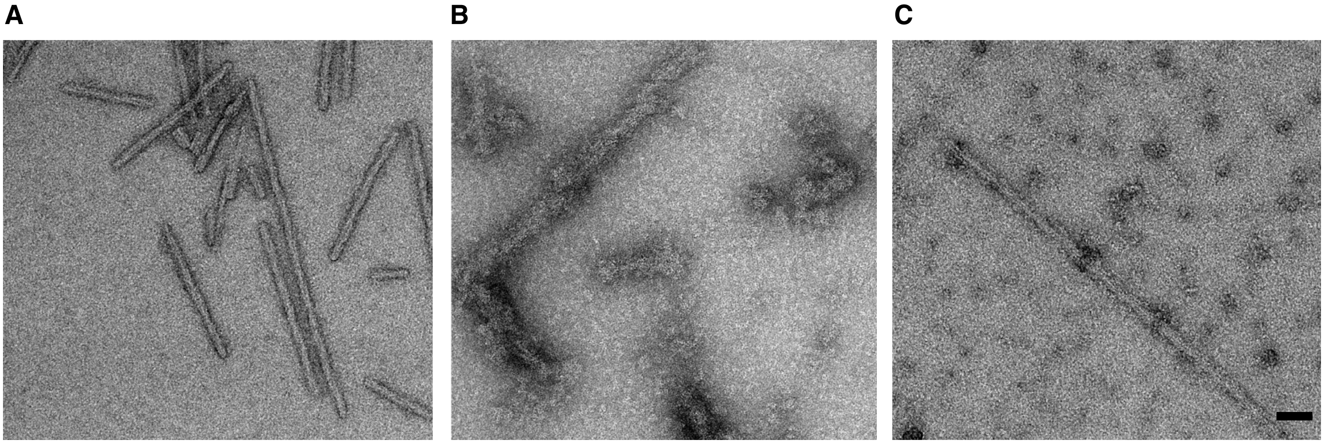

Figure EV4. Negative stain EM images showing that chaperone recruitment to fibres did not occur when fibres were incubated with Hsc70/ΔJ-DNAJB1/Apg2/ATP.

A–C In negative stain images, chaperone binding appeared as a pronounced increase in fibre thickness, visible when comparing images of αSyn samples alone to those incubated with Hsc70/DNAJB1/Apg2. (A) αSyn fibres alone; (B) fibres + Hsc70/DNAJB1/Apg2/ATP; (C) fibres + Hsc70/ΔJ-DNAJB1/Apg2/ATP. Scale bar, 50 nm.

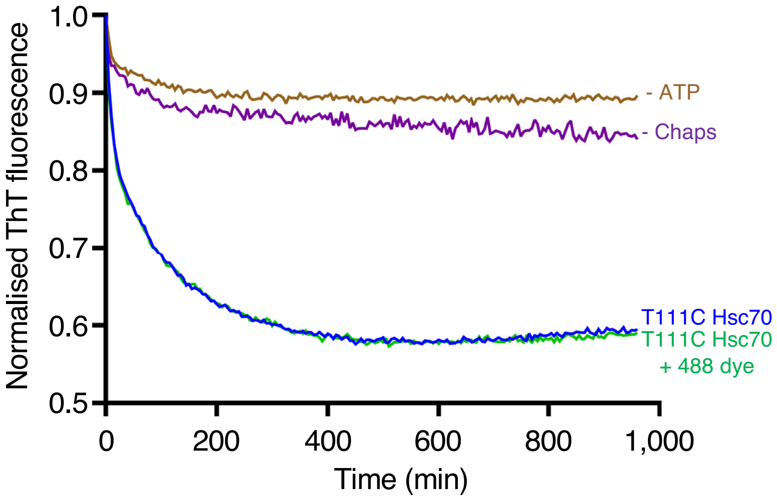

Figure EV5. Disaggregation activity measured by Thioflavin T fluorescence.

Labelling T111C Hsc70 mutant with the 488 maleimide dye does not affect the efficiency of the disaggregation.

© 2022 The Authors
