# Supplementary material for: Cooperative amyloid fibre binding and disassembly by the Hsp70 disaggregase
Source: EMBO J. 2022 Jun 13;41(16):e110410. doi: 10.15252/embj.2021110410 (PMC9379549; doi:10.15252/embj.2021110410)
Supplement: Supplementary file 2 — Movie EV1 [file EMBJ-41-e110410-s006.zip › Movie EV1.docx]

Movie EV1. AFM movie from Figure 1A.
