# Supplementary material for: Cooperative amyloid fibre binding and disassembly by the Hsp70 disaggregase
Source: EMBO J. 2022 Jun 13;41(16):e110410. doi: 10.15252/embj.2021110410 (PMC9379549; doi:10.15252/embj.2021110410)
Supplement: Supplementary file 3 — Movie EV2 [file EMBJ-41-e110410-s008.zip › Movie EV2.docx]

Movie EV2. AFM movie from Figure 1B.
