# Supplementary material for: Cooperative amyloid fibre binding and disassembly by the Hsp70 disaggregase
Source: EMBO J. 2022 Jun 13;41(16):e110410. doi: 10.15252/embj.2021110410 (PMC9379549; doi:10.15252/embj.2021110410)
Supplement: Supplementary file 4 — Movie EV3 [file EMBJ-41-e110410-s002.zip › Movie EV3.docx]

Movie EV3. Tomogram of αSyn fibres with DNAJB1, Hsc70 and ATP.
