# Supplementary material for: Cooperative amyloid fibre binding and disassembly by the Hsp70 disaggregase
Source: EMBO J. 2022 Jun 13;41(16):e110410. doi: 10.15252/embj.2021110410 (PMC9379549; doi:10.15252/embj.2021110410)
Supplement: Supplementary file 6 — Table EV1 [file EMBJ-41-e110410-s003.docx]

| Fibre number | Starting length (nm) | Final length (nm) |
| --- | --- | --- |
| 1 | 262 | 177 |
| 2 | 310 | 0 |
| 3 | 888 | 352 |
| 4 | 710 | 360 |
| 5 | 1200 | 968 |
| 6 | 237 | 0 |
| 7 | 374 | 57 |
| 8 | 359 | 0 |
| 9 | 415 | 0 |
| 10 | 556 | 0 |
| 11 | 337 | 230 |
| 12 | 417 | 195 |
| 13 | 152 | 0 |
| 14 | 231 | 0 |
| 15 | 173 | 0 |
| 16 | 1800 | 1380 |
| 17 | 1210 | 0 |

**Table EV1: The starting and final lengths of all αSyn fibres whose disassembly was visualised in AFM videos.**
