# Supplementary material for: Cooperative amyloid fibre binding and disassembly by the Hsp70 disaggregase
Source: EMBO J. 2022 Jun 13;41(16):e110410. doi: 10.15252/embj.2021110410 (PMC9379549; doi:10.15252/embj.2021110410)
Supplement: Supplementary file 7 — Table EV2 [file EMBJ-41-e110410-s004.docx]

| Buffer | Buffer Composition |
| --- | --- |
| HKMD buffer | 50 mM HEPES, 50 mM KCl, 5 mM MgCl_2_, 2 mM DTT, pH 7.50 |
| HKMT buffer | 50 mM HEPES, 50 mM KCl, 5 mM MgCl_2_, 2 mM TCEP, pH 7.50 |
| Disaggregation buffer | 50 mM HEPES, 50 mM KCl, 5 mM MgCl_2_, 2 mM DTT, pH 7.50 5 mM ATP, 6 mM PEP, 20 ng/µL pyruvate kinase |
| Hsp70/110 lysis buffer | 50 mM HEPES, 150 mM KCl, 5 mM MgCl_2_, 2 mM DTT, EDTA free protease inhibitor tablet (Roche), pH 7.50 |
| Hsp70/110 wash 1 buffer | 50 mM HEPES, 150 mM KCl, 5 mM MgCl_2_, 0.1 mM PMSF, 2 mM DTT, pH 7.50 |
| Hsp70/110 wash 2 buffer | 50 mM HEPES, 150 mM KCl, 5 mM MgCl_2_, 40 mM imidazole, 2 mM DTT, pH 7.50 |
| ATP wash buffer | 50 mM HEPES, 150 mM KCl, 5 mM MgCl_2_, 5mM ATP, 2 mM DTT, pH 7.50 |
| DNAJ lysis buffer | 50 mM HEPES, 750 mM KCl, 5 mM MgCl_2_, 10 % glycerol, pH 7.50 |
| DNAJ wash 1 buffer | 50 mM HEPES, 150 mM KCl, 5 mM MgCl_2_, 10 % glycerol, 40 mM Imidazole, pH 7.50 |
| DNAJ wash 2 buffer | 50 mM HEPES, 50 mM KCl, 5 mM MgCl_2_, 10 % glycerol, 40 mM, Imidazole, pH 7.50 |
| DNAJ elution buffer | 50 mM HEPES, 750 mM KCl, 5 mM MgCl_2,_ 10 % glycerol, 500 mM Imidazole, pH 7.50 |
| DNAJ SEC buffer | 50 mM HEPES, 750 mM KCl, 5 mM MgCl_2_, 10 % glycerol, pH 7.50 |
| αSyn lysis buffer | 100 mM Tris-HCl, 10 mM EDTA, 2 mM DTT, pH 8.0 |
| αSyn buffer A | 25 mM Tris-HCl, 2 mM DTT, pH 7.7 |
| αSyn buffer B | 25 mM Tris-HCl, 1 M NaCl, 2 mM DTT, pH 7.7 |
| αSyn fibrillation buffer | 50 mM NaPO_4_, 100 mM NaCl, 0.05% w/v NaN_3_, pH 7.30 |
| TBS Buffer | 50 mM Tris-HCl, 150 mM NaCl, pH 7.50 |
| HEPES Buffer | 50 mM HEPES, 2mM DTT, pH 7.50 |

**Table EV2. The buffers used for protein purification and amyloid fibre disaggregation reactions**.
