# Supplementary material for: Cooperative amyloid fibre binding and disassembly by the Hsp70 disaggregase
Source: EMBO J. 2022 Jun 13;41(16):e110410. doi: 10.15252/embj.2021110410 (PMC9379549; doi:10.15252/embj.2021110410)
Supplement: Supplementary file 8 [file EMBJ-41-e110410-s005.pdf]

THE EMBO JOURNAL

Cooperative Amyloid Fibre Binding and Disassembly by the Hsp70 disaggregase

Joseph Beton, Jim Monistrol, Anne Wentink, Erin Johnston, Anthony Roberts, Bernd Bukau, Bart Hoogenboom, and Helen Saibil

DOI: 10.15252/embj.2021110410

Corresponding author(s): Helen Saibil (h.saibil@mail.cryst.bbk.ac.uk)

Review Timeline:

Submission Date:	10th Dec 21
Editorial Decision:	31st Jan 22
Revision Received:	5th Apr 22
Editorial Decision:	28th Apr 22
Revision Received:	3rd May 22
Accepted:	6th May 22

Editor: Stefanie Boehm

Transaction Report:

(Note: With the exception of the correction of typographical or spelling errors that could be a source of ambiguity, letters and reports are not edited. Depending on transfer agreements, referee reports obtained elsewhere may or may not be included in this compilation. Referee reports are anonymous unless the Referee chooses to sign their reports.)

1st Editorial Decision **31st Jan 2022**

Thank you for submitting your manuscript on the mechanisms of a-synuclein fiber disassembly to The EMBO Journal. We have now received three referee reports on your study, which are included below for your information. In light of the referees' comments, we would like to invite you to prepare and submit a revised manuscript.

As you will see, the reviewers are overall positive and appreciate the findings. However, they also raise a number of points that should be addressed before the study is considered further for publication. In particular, referees #2 and #3 find that the link between fragmentation and depolymerization would need to be strengthened (ref#2- point 1) and the role of Apg2 further defined (ref #3). In addition, referee #2 is concerned that a physical interaction of the chaperone complex with the fibril could also be responsible for disassembly (point 2). Please address these concerns by adding further experimental data or, if appropriate, through additional or revised discussion of the specific issues in the manuscript. Please also carefully consider all other referee comments and revise the manuscript and figures as needed, as well as providing a detailed response to each comment.

Please note that it is our policy to allow only a single round of major revision. Acceptance depends on a positive outcome of a second round of review and therefore on the completeness of your responses included in the next, final version of the manuscript. I encourage you to review the referees' comments and contact me to discuss a preliminary revision plan in case there are any uncertainties regarding specific points or in general.

Referee #1:

The manuscript by Beton et al. describes elegant studies of alpha-synuclein fibril disaggregation by the Hsp70 system. This study relies on powerful AFM methods that enabled the authors to observe disaggregation in real time under a variety of conditions in which the stoichiometry and component participation of the Hsp70 system are varied. The results are breath-taking, rigorously analyzed, and compellingly interpreted. The paper is essentially acceptable for publication in its current form. I have only a few minor points that the authors may wish to address:

1. A paper that was published on this same system by M. Schneider et al., Nat. Comm. 12, 5999 (2021) is not cited or mentioned, most likely because it came out after this paper was submitted. In any case, it should be cited.
2. The model proposed by these authors seems to differ slightly from one proposed in Wentik et al. in their recent paper; the role of Apg2 seems somewhat different. Please be sure to reconcile these models, and if the role of Apg2 is intended to be different, make this clear.
3. Figure 2E is difficult to understand and the reader would benefit from a better explanation.

Referee #2:

Highly stable protein aggregates, also known as amyloid fibres, can be disassembled by chaperones. The human Hsc70 disaggregase, composed of Hsc70, DNAJB1, and Apg2, has been shown to mediate such disassembly but the dynamics of this activity is still unclear. Using a-synuclein protein fibres as a model, Beton et al., visualize at the nanoscale, how Hsc70, DNAJB1 and Apg2 work together to depolymerize fibres. They find that disassembly occurs at fibril ends and progresses in one direction along the fibre. Depolymerization appears to occur in short bursts, rather than by a gradual dissociation, which takes place at fibre sites that are densely bound by chaperones. The nuclear exchange factor (NEF) Apg2 increases the density of chaperones in a clusters, which is accompanied by enhanced depolymerisation. By visualizing the chaperone dynamics at the nanoscale, the authors reveal unexpected aspects of depolymerisation, such as the importance of fragmentation and local chaperone clustering. Knowing these dynamic properties are important if one would like explore intervention to dissociate fibrils as a treatment for amyloid diseases. While most data are solid and support the main conclusions, some parts need more clarification. In particular the links between fragmentation and depolymerisation as well as the distinction between chaperone binding and disaggregase activity are not clear. See below for more specific comments.
Major comments:

1. The link between fragmentation and disassembly is not clearly described. Is the chaperone complex also present at the site of fragmentation, before fragmentation occurs? Does fragmentation precede disassembly at the same position? If so, does the complex stay on one site of the two fragments or on both? Do you also find disassembly at the original fiber ends that have not been fragmented?
2. The results do not exclude the possibility that a physical interaction between fibril and chaperone complex, rather than its disaggregase activity, is responsible for disassembly. How can you distinguish between the requirements for binding and those for disassembly? Would it be possible for the authors to include a condition that still shows dense binding of complete but inactive Hsc70/DNAJB1/Apg2 complexes, e.g. with inactive mutants of Hsc70 or Apg2? Current controls, including a Hsc70-non-binding DNAJB1, seem all to involve incomplete or non-binding disaggregase complexes.
3. Figure 3. Not sure how to see the difference here between B and C, please indicate what is counted.
4. Figure 2C? Why are not data of all 22 fibers included, please include.

5. How has Figure 3 been quantified? This is not described in the M&M sections? Are the fibrils blinded in this case. A better description of procedure used is needed. How do the authors differentiate between full and end. What defines a dense decoration?

Minor comments
6. If depolymerisation is determined by structural polarity, what determines this polarity? How is it recognized?
7. Are the labeled Hsc70 molecules use for TIRF also active in disassembly? Such evidence would support the connection between the localization of the complex on the fiber and their activity as disassemblers.
8. The authors show that some fibrils are bound by the chaperone complex and others are not. Can the authors think of an explanation? Are intact fibrils unaffected and unbound? How are the original fiber ends doing in comparison to the break points of fibrils with respect to complex binding and depolymerization?
9. The authors write: "The helical twist can be detected in the AFM experiments prior to chaperone injection (Figure 1A,B). Since no such correlation is observed, we rule out such an artefact (Supplementary Figure 2). " why is this an argument to exclude an artifact from adhesion?
10. Can the authors compare the fragment types and sizes after fragmentation with those in an in vitro reaction, do they correspond?
11. Is the increase in height due a consequence of fragmentation or the fact that chaperones collect at these regions? Could it be that the loosening up is first and then the chaperones can collect at a certain site? Can the authors experimentally distinguish between these options? These height changes are not seen before fragmentation. Does this mean that chaperones are not present or that they do not result in an increase in height? Do breaks also occur without chaperones?
12. How does the length of the densely packed chaperone clusters correspond to the length of the disassembled region?
13. Agree with the author that "It is surprising that a recycling factor (NEF), which is expected to release Hsc70 from its substrate, increases total Hsc70 bound to the fibrils. "How do you explain this observation? Would a mutant hsc70 that is insensitive to apg2 have the same effect? Is binding of Apg2 or its NEF function required for the observed binding? Can the authors experimentally distinguish between these options? See also comment 2.
14. How can dense binding cause a burst of disassembly rather than a gradual one? Can the authors zoom into the burst time windows at higher resolution with AFM to exclude a gradual decrease?

Referee #3:

In this paper, the authors visualized the disaggregation process of Hsp70-mediated alpha-syn fibers using high-speed AFM and cryo-EM, and investigated the contribution of DNAJB1 and APG2 in detail. They found that the degradation proceeded in a unidirectional manner. We also found that APG2 strongly promoted the recruitment of Hsc70. The research design is adequate, the methods used for this study are solid and data obtained are solid. I have several comments and suggestions for the authors to consider improving the quality of the paper.

1. Visualization of Apg2-mediated Hsc70 recruitment by AFM
The authors hypothesized that the recruitment of Hsc70/DNAJB1/Apg2 to fibers induces a large cluster that attributes the ~5 nm change in fiber height. Although they showed the increase in the decoration at the fiber ends by cryo-EM, it would be intriguing to analyze the height of fibers with Hsc70/DNAJB1 to ascertain that the height change is dependent on Apg2-mediated Hsc70 recruitment. In fact, Apg2 is an enhancing, but not essential, factor for disaggregation of alpha-syn fiber in their assay (Fig. 5D, "-apg2"). Thus, a comparison of the fibers with Hsc70/DNAJB1/Apg2 and Hsc70/DNAJB1 provides strong evidence that the height change is related to the accerralated disaggregation mechanism.

2. Dependence of Apg2 on the disaggregation burst
One of the intriguing points of this paper is that the alpha-syn fibers were disaggregated in rapid processive bursts. However, it remains unclear whether this disaggregation burst is dependent on the Apg2-mediated Hsc70 recruitment, as the alpha-syn fiber is depolymerized by Hsc70/DNAJB1. Again, kinetic analysis of the disaggregation process of the alpha-syn with Hsc70/DNAJB1 would provide a further precise understanding of the role of Apg2.

3. Binding of Apg2 to the alpha-syn fibers
As the authors indicated, Apg2 was not visible in the CBB stained pellet fractions. The previous paper (Gao et al., 2015) showed the irregularly binding of Apg2 on the fiber by tomography, it remains unclear whether Apg2 is recruited to the Hsc/DNAJB1 complex to the fiber as hypothesized in fig. 7. Thus the reviewer recommends performing immunoelectron microscopic analysis of the fiber with Hsc70/DNAJB1/Apg2 complex to visualize the existence of functional Apg2 on the alpha-syn fiber.

EMBOJ-2021-110410
Cooperative Amyloid Fibre Binding and Disassembly by the Hsp70 disaggregase

Reply to reviewers

Referee #1:

The manuscript by Beton et al. describes elegant studies of alpha-synuclein fibril disaggregation by the Hsp70 system. This study relies on powerful AFM methods that enabled the authors to observe disaggregation in real time under a variety of conditions in which the stoichiometry and component participation of the Hsp70 system are varied. The results are breath-taking, rigorously analyzed, and compellingly interpreted. The paper is essentially acceptable for publication in its current form. I have only a few minor points that the authors may wish to address:

1. A paper that was published on this same system by M. Schneider et al., Nat. Comm. 12, 5999 (2021) is not cited or mentioned, most likely because it came out after this paper was submitted. In any case, it should be cited.

We thank the reviewer for pointing this out. In this revised submission, we have included this reference as well as another very relevant paper that appeared after our initial submission (Franco et al, PNAS 2021 118 (36) e2105548118). Both papers include data that are consistent with aspects revealed in our study and provide complementary information.
These papers are now cited in the introduction and discussion.

2. The model proposed by these authors seems to differ slightly from one proposed in Wentik et al. in their recent paper; the role of Apg2 seems somewhat different. Please be sure to reconcile these models, and if the role of Apg2 is intended to be different, make this clear.

The FRET experiments of Wentink et al were not designed to look for increased Hsp70 loading, and the increase in recruitment was not detectable by fluorescence anisotropy because of the high background of unbound protein. The background signal had less impact in our binding assays and TIRF experiments. Our new findings reveal that Apg2 increases the recruitment of Hsc70 (Fig. 5), a role that was not previously reported.

This point has been clarified in the discussion.

3. Figure 2E is difficult to understand and the reader would benefit from a better explanation.

Figure 2E shows height profiles of a fibre for different time points. The caption of this figure has been revised to make this clearer.
Fig 2E caption has been revised.

Referee #2:

Highly stable protein aggregates, also known as amyloid fibres, can be disassembled by chaperones. The human Hsc70 disaggregase, composed of Hsc70, DNAJB1, and Apg2, has been shown to mediate such disassembly but the dynamics of this activity is still unclear.

Using a-synuclein protein fibres as a model, Beton et al., visualize at the nanoscale, how Hsc70, DNAJB1 and Apg2 work together to depolymerize fibres. They find that disassembly occurs at fibril ends and progresses in one direction along the fibre. Depolymerization appears to occur in short bursts, rather than by a gradual dissociation, which takes place at fibre sites that are densely bound by chaperones. The nuclear exchange factor (NEF) Apg2 increases the density of chaperones in a clusters, which is accompanied by enhanced depolymerisation. By visualizing the chaperone dynamics at the nanoscale, the authors reveal unexpected aspects of depolymerisation, such as the importance of fragmentation and local chaperone clustering. Knowing these dynamic properties are important if one would like explore intervention to dissociate fibrils as a treatment for amyloid diseases. While most data are solid and support the main conclusions, some parts need more clarification. In particular the links between fragmentation and depolymerisation as well as the distinction between chaperone binding and disaggregase activity are not clear. See below for more specific comments.

Major comments:

1. The link between fragmentation and disassembly is not clearly described. Is the chaperone complex also present at the site of fragmentation, before fragmentation occurs? Does fragmentation precede disassembly at the same position? If so, does the complex stay on one site of the two fragments or on both? Do you also find disassembly at the original fiber ends that have not been fragmented?

From our data, we conclude that fibre disaggregation is preceded by local chaperone binding and predominantly occurs from one fibre end. It is correct that most of the disassembly events we observe are at the “original fibre ends that have not been fragmented”. By contrast, fragmentation is observed/defined as breaks within the fibre.
Fragmentation is a less common event in our experiments (5 breaks detected in 16 hours, 40 minutes of video (not including negative controls)). It is therefore not possible to analyse them in detail. We did not detect any correlation between fragmentation and depolymerisation, except for the fragmentation resulting in fibre ends from which depolymerisation could be initiated. That is, fragmentation could contribute to disaggregation by providing new sites for initiation/nucleation of the disaggregation reaction. It is also a potential source of fibril seeds that can be trafficked between cells so could be important in a disease context. In the example shown, there is a height increase “downstream” of the break, i.e. consistent with the polarity of depolymerisation.
We note that fragmentation was not reported by either of the recent papers mentioned in reply 1 above.

Some of these points have been added to Results and discussion sections, pages 6 and 14.

2. The results do not exclude the possibility that a physical interaction between fibril and chaperone complex, rather than its disaggregase activity, is responsible for disassembly. How can you distinguish between the requirements for binding and those for disassembly? Would it be possible for the authors to include a condition that still shows dense binding of complete but inactive Hsc70/DNAJB1/Apg2 complexes, e.g. with inactive mutants of Hsc70 or Apg2? Current controls, including a Hsc70-non-binding DNAJB1, seem all to involve incomplete or non-binding disaggregase complexes.

The reviewer appears to ask if there is a way by which enzymatically inactive mutant chaperones could bind at similar density to the fibre without causing disassembly. According to the entropic pulling model, it is the binding that causes the pulling, not a particular conformational change or [chemical?] activity of Hsp70. It is just that the dense binding required for disaggregation can only be achieved (on a physiological timescale) through the selective recycling activity of Apg2.

In all our (and others') experiments so far, chaperone binding and disaggregation activity are tightly correlated (see, e.g., Fig. 5F). However, Faust et al (2020) report that the ΔH5 mutant of DNAJB1, which is defective in autoinhibition, supports Hsp70 binding, albeit not appearing to lead to similar levels of clustering on the fibres and thereby showing little disaggregation. The autoinhibitory action of DNAJB1 is required for disaggregation, but the mechanism is not currently understood. At least in the case of this DNAJB1 mutant, binding is not sufficient for disaggregation. A detailed characterisation of this binding will require an extensive structural and statistical study and is beyond the scope of this paper.

Discussion text modified to point out that binding is necessary but not sufficient for disaggregation.

3. Figure 3. Not sure how to see the difference here between B and C, please indicate what is counted.

Prompted by this question and q5 below, a different author did a new blinded count of these images, and we have concluded that it is too difficult to reliably discern the differences. We have removed the counting of decoration on cryo EM images. The increase in Hsc70 recruitment is best shown by the binding assays and TIRF, and the binding difference between the two ends of a fibre is best shown by TIRF and AFM.

Figure 3, methods and results text have been modified.

4. Figure 2C? Why are not data of all 22 fibers included, please include.

Figure 2C includes >10 disaggregation events (sharp drops in fibre length as a function of time). For the more comprehensive data set, we refer to the various AFM movies included in this submission. We did not include more length-vs-time profiles in Figure 2C because this would compromise the readability of this figure as shown below, hindering its purpose of illustrating/clarifying the sharp, burst-like activity that is also apparent from inspection of the various AFM movies.

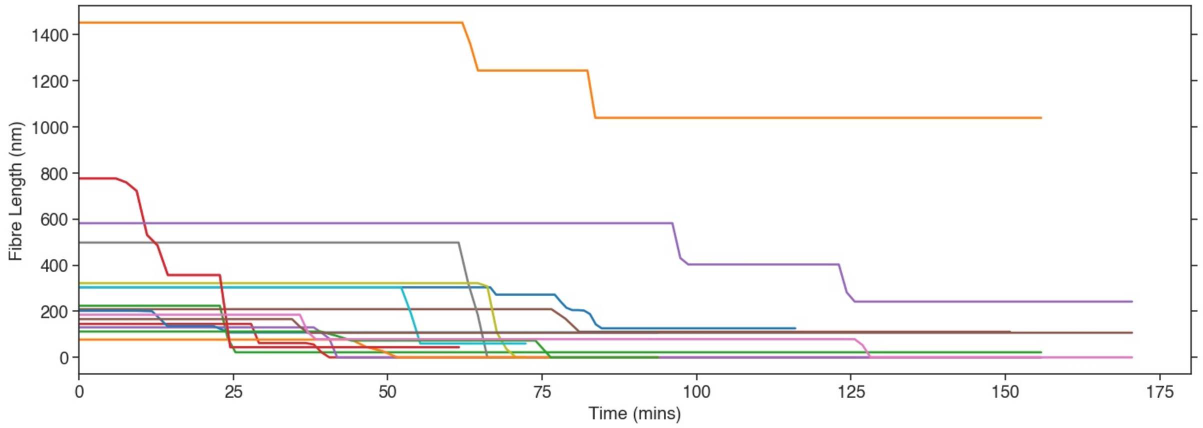

5. How has Figure 3 been quantified? This is not described in the M&M sections? Are the fibrils blinded in this case. A better description of procedure used is needed. How do the authors differentiate between full and end. What defines a dense decoration?

Please see the reply to point 3.

Minor comments

6. If depolymerisation is determined by structural polarity, what determines this polarity? How is it recognized?

Since the structure has polarity, the surfaces exposed by the subunits at the two ends of the fibre are different. It is likely that one end is less stable than the other, and/or more accessible to chaperone engagement. A key Hsc70 binding site lies in the core of the amyloid fold (αsyn residues 37-43, Wentink et al, 2020). At this site, the two ends of the polar αsyn structure show a consistent difference in positive charge, but no significant differences in hydrophobicity, for all the known fibre structures of unmodified αsyn. Therefore, we can speculate that chaperone action is polar due to the difference in surface charge at the two fibre ends.

We have added figure 6 to illustrate this point for PDB 6CU7, with more examples shown here.

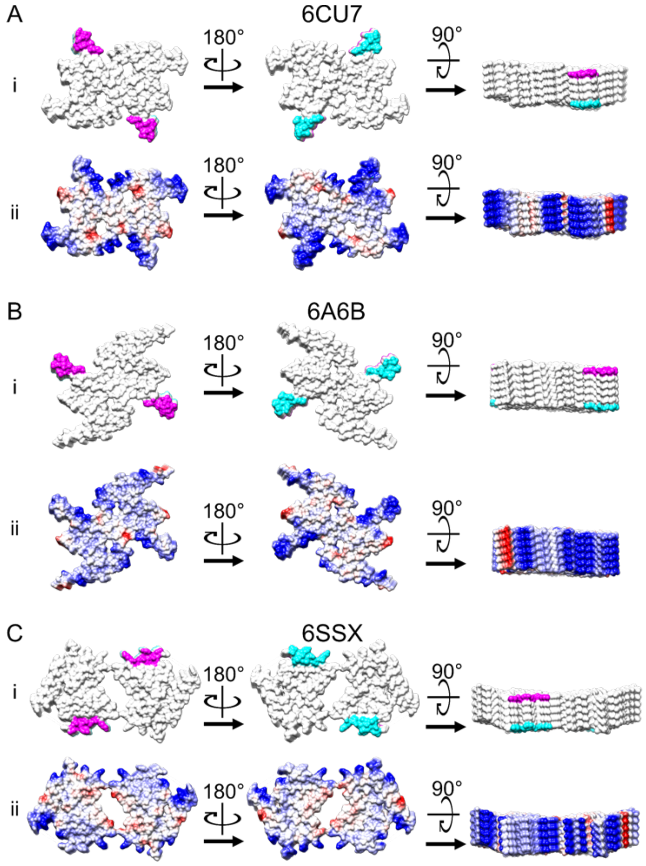

The polarity of αSyn amyloid fibrils. Top, bottom and side views of rod (A, PDB: 6CU7), type 1b (B, 6A6B) type 2a (C, PDB: 6SSX) conformers. Panels (i) highlight the second binding sites for Hsc70 (residues 37-43) in magenta and cyan at the two ends of the fibre. Panels (ii) display the electrostatic surfaces of the different views. Negatively charged residues are shown in red, positively charged residues in blue. The electrostatic surfaces indicate that the top and bottom surfaces expose different amounts of positive charge at the 37-43 binding site for Hsc70 on αSyn.

7. Are the labeled Hsc70 molecules use for TIRF also active in disassembly? Such evidence would support the connection between the localization of the complex on the fiber and their activity as disassemblers.

The activity was checked with a ThT assay (done in triplicate) using the labelled Hsc70, which shows that disassembly activity is not affected by the Alexa488 label.

Shown as new extended view figure 5.

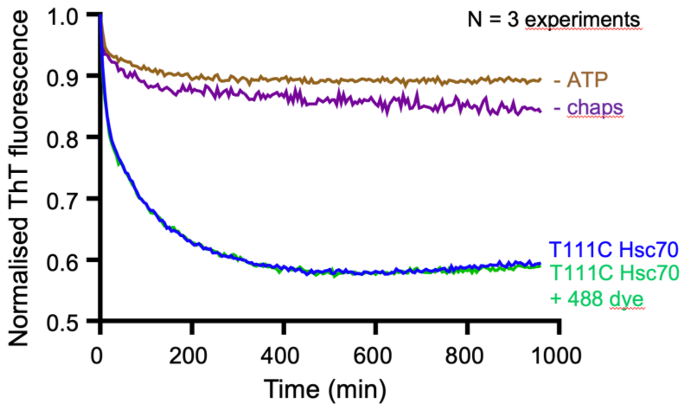

8. The authors show that some fibrils are bound by the chaperone complex and others are not. Can the authors think of an explanation? Are intact fibrils unaffected and unbound? How are the original fiber ends doing in comparison to the break points of fibrils with respect to complex binding and depolymerization?

In experiments distinguishing individual fibres on the nanometre length scale, we expect to see stochastic binding, further enhanced by the cooperative recruitment that we report on. Once there is stochastic local nucleation of chaperone binding, this enhances further binding, resulting in wide variations in chaperone binding to different fibres and to different locations at the same fibre, as observed. Apg2 does not promote nucleation (around 2/3 of the fibres are decorated, +/- Apg2). The cooperativity would explain why dense clusters of chaperones are formed rather than uniform decoration along the fibre.

We cannot formally exclude that we have a mixture of different fibre polymorphs with different chaperone interactions. These could be detected by high resolution cryo EM but not by AFM, so they cannot be correlated with depolymerisation. However, all the fibre types we have detected are capable of binding DNAJB1.

The second part of the question seems to imply that perhaps the fibril ends formed by fragmentation are not the same as those of the original fibril. We have not seen any evidence for this – EM of the fibres shows blunt ends under all the conditions we have used.

9. The authors write: "The helical twist can be detected in the AFM experiments prior to chaperone injection (Figure 1A,B). Since no such correlation is observed, we rule out such an artefact (Supplementary Figure 2). " why is this an argument to exclude an artifact from adhesion?

We have tried to better clarify that a helical track will periodically pass through the underside of the fiber, ie next to the surface support that might protect the fibre from disassembly. In other words, if the surface support/adhesion were a significant factor in arresting depolymerisation, we would expect a correlation between removed fibre lengths and helical periodicity. Such a correlation is not observed. This is also described in further detail in the caption of extended view Fig. 2.

Point clarified in the results, page 6.

10. Can the authors compare the fragment types and sizes after fragmentation with those in an in vitro reaction, do they correspond?

All our experiments represent *in vitro* reactions. We are unsure if the reviewer is referring to the difference between a disaggregation reaction in the test tube (which contains free fibrils in bulk solution) vs the AFM data (which detects individual fibres immobilised on a surface); if so the two experiments are not directly comparable.

11. Is the increase in height due a consequence of fragmentation or the fact that chaperones collect at these regions? Could it be that the loosening up is first and then the chaperones can collect at a certain site? Can the authors experimentally distinguish between these options? These height changes are not seen before fragmentation. Does this mean that chaperones are not present or that they do not result in an increase in height? Do breaks also occur without chaperones?

The EM and TIRF confirm the chaperone binding, which corresponds to the conditions of height increase. There is no Hsc70 binding, disaggregation, fragmentation or height increase in the absence of chaperones and ATP (fig EV1).
There is one example (figure 1B) where a local height increase doesn't lead to disaggregation and one example that shows "spontaneous" disaggregation, i.e., with no preceding height increase (figure 1A, figure 2A). In the other 24 cases, there was excellent correlation.

12. How does the length of the densely packed chaperone clusters correspond to the length of the disassembled region?

Fig 2E shows an excellent correspondence for one example. The average length of fibre depolymerised in a single burst (176 ± 84 nm) corresponds very closely to the length of a raised segment, which we interpret to be densely packed chaperones, as measured by AFM (178 ± 65).

This point has been added to results, page 8.

13. Agree with the author that "It is surprising that a recycling factor (NEF), which is expected to release Hsc70 from its substrate, increases total Hsc70 bound to the fibrils. "How do you explain this observation? Would a mutant hsc70 that is insensitive to apg2 have the same effect? Is binding of Apg2 or its NEF function required for the observed binding? Can the authors experimentally distinguish between these options? See also comment 2.

It seems that the recycling activity of the Apg2 promotes the dense clustering of Hsc70 at a fibre end. The interaction between Apg2 and the fibre is very weak and is not needed to explain the observations. Disaggregation does not depend on the ATPase activity of Apg2 but NEF activity is essential (based on NEF deficient mutant A279A, N280A, N619Y, E622A). In addition, Wentink et al (2020) showed that other NEFs such as Bag1 can more effectively support disaggregation if they are increased in size by attachment of bulky components.

14. How can dense binding cause a burst of disassembly rather than a gradual one? Can

the authors zoom into the burst time windows at higher resolution with AFM to exclude a gradual decrease?

It is true that the description of a “burst-like” or “gradual” disassembly depends on the timescale of the measurement (since any burst will appear gradual when considered at sufficiently high temporal resolution). For clarification, our point is that the disaggregation reaction appears to *propagate* along the fibre much faster (“burst-like” and “cooperatively”) than the timescale at which this reaction is *initiated*, and this is very clear within the resolution of the AFM data included in our manuscript. The AFM videos recorded by Franco et al had faster time resolution and the fibre shortening speed appeared 2-4x faster than our estimate, which is a reasonable agreement given the multiple differences in experimental conditions, chaperone concentrations, etc.

Referee #3:

In this paper, the authors visualized the disaggregation process of Hsp70-mediated alpha-syn fibers using high-speed AFM and cryo-EM, and investigated the contribution of DNAJB1 and APG2 in detail. They found that the degradation proceeded in a unidirectional manner. We also found that APG2 strongly promoted the recruitment of Hsc70. The research design is adequate, the methods used for this study are solid and data obtained are solid. I have several comments and suggestions for the authors to consider improving the quality of the paper.

1. Visualization of Apg2-mediated Hsc70 recruitment by AFM
The authors hypothesized that the recruitment of Hsc70/DNAJB1/Apg2 to fibers induces a large cluster that attributes the ~5 nm change in fiber height. Although they showed the increase in the decoration at the fiber ends by cryo-EM, it would be intriguing to analyze the height of fibers with Hsc70/DNAJB1 to ascertain that the height change is dependent on Apg2-mediated Hsc70 recruitment. In fact, Apg2 is an enhancing, but not essential, factor for disaggregation of alpha-syn fiber in their assay (Fig. 5D, "-apg2"). Thus, a comparison of the fibers with Hsc70/DNAJB1/Apg2 and Hsc70/DNAJB1 provides strong evidence that the height change is related to the accerralated disaggregation mechanism.

In principle this is an excellent experiment, but in practice, such a comparison is hampered by the low throughput of the AFM experiments. In the presence of Hsc70/DNAJB1/Apg2 (+ATP), we report on 26 depolymerisation events from 17 fibres in 16+ hours of video recording. Without Apg2, we may expect (based on Fig. 5D-E and previous studies) the number of depolymerisation events to be 3-4x lower, making it hard to obtain statistically robust results.

2. Dependence of Apg2 on the disaggregation burst
One of the intriguing points of this paper is that the alpha-syn fibers were disaggregated in rapid processive bursts. However, it remains unclear whether this disaggregation burst is dependent on the Apg2-mediated Hsc70 recruitment, as the alpha-syn fiber is depolymerized by Hsc70/DNAJB1. Again, kinetic analysis of the disaggregation process of the alpha-syn with Hsc70/DNAJB1 would provide a further precise understanding of the role of Apg2.

While DNAJB1 and Hsc70 are indeed the main constituents of the observed chaperone clusters, disaggregation is extremely inefficient in the absence of Apg2. As indicated above, the suggested kinetic analysis is thus not feasible. However, the correlation of activity with

specific, Apg2 dependent Hsc70 recruitment to the end clusters is well supported by the binding, TIRF and tomography experiments.

3. Binding of Apg2 to the alpha-syn fibers
As the authors indicated, Apg2 was not visible in the CBB stained pellet fractions. The previous paper (Gao et al., 2015) showed the irregularly binding of Apg2 on the fiber by tomography, it remains unclear whether Apg2 is recruited to the Hsc/DNAJB1 complex to the fiber as hypothesized in fig. 7. Thus the reviewer recommends performing immunoelectron microscopic analysis of the fiber with Hsc70/DNAJB1/Apg2 complex to visualize the existence of functional Apg2 on the alpha-syn fiber.

In the Gao et al paper, immunogold EM was done but it was not possible to establish if the gold labelling coincided with chaperone clusters. In the light of more recent work, it seems that the role of Apg2 is to recycle Hsc70 and Apg2 binding to the fibres is weak and transient.
We tried to detect Apg2 by fluorescence but the signal was too weak. This is not surprising, since it only binds fibres at 1/10th the level of Hsc70, in equilibrium with unbound Apg2.

In fact, the key role of Apg2 may be to stimulate Hsc70 recycling and clustering once Hsc70 has bound to the more susceptible/less stable fibre end. A new figure showing the difference in charge at that binding site between the two ends of the fibre has been added (referee 2 q6), and the final diagram has been modified to make this point. This mechanism does not require Apg2 binding all along the fibre, only a transient interaction at one end.

Thank you again for submitting your revised manuscript. We have now received comments from the initial three referees (please see below) and I am pleased to say that they now support publication. Thus I would now like to ask you to resolve a number of editorial issues that are listed in detail below. Please use the document that the data editors have added their comments to for any changes (see below). Please feel free to contact me if you have further questions regarding the revision or any of the specific points. Thank you again for giving us the chance to consider your manuscript for The EMBO Journal.

Referee #1:

The comments I made in my review of the earlier version of this manuscsript have been satisfactorily addressed.

Referee #2:

All concerns have been addressed well.

Referee #3:

Authors fulfilled our request.

The authors have made all requested editorial changes.

2nd Revision - Editorial Decision **6th May 2022**

Thank you again for submitting the final revised version of your manuscript and addressing the remaining points. I am pleased to inform you that we have now accepted it for publication in The EMBO Journal.

EMBO Press Author Checklist

Corresponding Author Name: Helen Saibil	
Journal Submitted to: EMBO J	
Manuscript Number: EMBOJ-2021-110410	

USEFUL LINKS FOR COMPLETING THIS FORM
The EMBO Journal - Author Guidelines
EMBO Reports - Author Guidelines
Molecular Systems Biology - Author Guidelines
EMBO Molecular Medicine - Author Guidelines

Reporting Checklist for Life Science Articles (updated January 2022)
This checklist is adapted from Materials Design Analysis Reporting (MDAR) Checklist for Authors. MDAR establishes a minimum set of requirements in transparent reporting in the life sciences (see Statement of Task: 10.31222/osf.io/9sm4x). Please follow the journal's guidelines in preparing your manuscript.
Please note that a copy of this checklist will be published alongside your article.

Abridged guidelines for figures
1. Data
The data shown in figures should satisfy the following conditions:
- the data were obtained and processed according to the field's best practice and are presented to reflect the results of the experiments in an accurate and unbiased manner.
- ideally, figure panels should include only measurements that are directly comparable to each other and obtained with the same assay.
- plots include clearly labeled error bars for independent experiments and sample sizes. Unless justified, error bars should not be shown for technical replicates.
- if n<5, the individual data points from each experiment should be plotted. Any statistical test employed should be justified.
- Source Data should be included to report the data underlying figures according to the guidelines set out in the authorship guidelines on Data Presentation.

2. Captions
Each figure caption should contain the following information, for each panel where they are relevant:
- a specification of the experimental system investigated (eg cell line, species name).
- the assay(s) and method(s) used to carry out the reported observations and measurements.
- an explicit mention of the biological and chemical entity(ies) that are being measured.
- an explicit mention of the biological and chemical entity(ies) that are altered/varied/perturbed in a controlled manner.
- the exact sample size (n) for each experimental group/condition, given as a number, not a range;
- a description of the sample collection allowing the reader to understand whether the samples represent technical or biological replicates (including how many animals, litters, cultures, etc.).
- a statement of how many times the experiment shown was independently replicated in the laboratory.
- definitions of statistical methods and measures:
  - common tests, such as t-test (please specify whether paired vs. unpaired), simple $\chi 2$ tests, Wilcoxon and Mann-Whitney tests, can be unambiguously identified by name only, but more complex techniques should be described in the methods section;
  - are tests one-sided or two-sided?
  - are there adjustments for multiple comparisons?
  - exact statistical test results, e.g., P values = x but not P values < x;
  - definition of 'center values' as median or average;
  - definition of error bars as s.d. or s.e.m.

Please complete ALL of the questions below.
Select "Not Applicable" only when the requested information is not relevant for your study.

Materials

Newly Created Materials	Information included in the manuscript?	In which section is the information available? (Reagents and Tools Table, Materials and Methods, Figures, Data Availability Section)
New materials and reagents need to be available; do any restrictions apply?	Yes	Materials and methods

Antibodies	Information included in the manuscript?	In which section is the information available? (Reagents and Tools Table, Materials and Methods, Figures, Data Availability Section)
For **antibodies** provide the following information:<br>- Commercial antibodies: RRID (if possible) or supplier name, catalogue number and or/clone number<br>- Non-commercial: RRID or citation	Not Applicable	

DNA and RNA sequences	Information included in the manuscript?	In which section is the information available? (Reagents and Tools Table, Materials and Methods, Figures, Data Availability Section)
**Short novel DNA or RNA including primers, probes**: provide the sequences.	Not Applicable	

Cell materials	Information included in the manuscript?	In which section is the information available? (Reagents and Tools Table, Materials and Methods, Figures, Data Availability Section)
**Cell lines:** Provide species information, strain. Provide accession number in repository **OR** supplier name, catalog number, clone number, and/**OR** RRID.	Not Applicable	
**Primary cultures:** Provide species, strain, sex of origin, genetic modification status.	Not Applicable	
Report if the cell lines were recently **authenticated** (e.g., by STR profiling) and tested for mycoplasma contamination.	Not Applicable	

Experimental animals	Information included in the manuscript?	In which section is the information available? (Reagents and Tools Table, Materials and Methods, Figures, Data Availability Section)
**Laboratory animals or Model organisms:** Provide species, strain, sex, age, genetic modification status. Provide accession number in repository **OR** supplier name, catalog number, clone number, **OR** RRID.	Not Applicable	
**Animal observed in or captured from the field:** Provide species, sex, and age where possible.	Not Applicable	
Please detail **housing and husbandry conditions**.	Not Applicable	

Plants and microbes	Information included in the manuscript?	In which section is the information available? (Reagents and Tools Table, Materials and Methods, Figures, Data Availability Section)
**Plants:** provide species and strain, ecotype and cultivar where relevant, unique accession number if available, and source (including location for collected wild specimens).	Not Applicable	
**Microbes:** provide species and strain, unique accession number if available, and source.	Not Applicable	

Human research participants	Information included in the manuscript?	In which section is the information available? (Reagents and Tools Table, Materials and Methods, Figures, Data Availability Section)
If collected and within the bounds of privacy constraints report on age, sex and gender or ethnicity for all study participants.	Not Applicable	

Core facilities	Information included in the manuscript?	In which section is the information available? (Reagents and Tools Table, Materials and Methods, Figures, Data Availability Section)
If your work benefited from core facilities, was their service mentioned in the acknowledgments section?	Yes	Materials and methods
	Select response	

Design

Study protocol	Information included in the manuscript?	In which section is the information available? (Reagents and Tools Table, Materials and Methods, Figures, Data Availability Section)
If study protocol has been **pre-registered, provide DOI in the manuscript**. For clinical trials, provide the trial registration number **OR** cite DOI.	Not Applicable	
Report the **clinical trial registration number** (at ClinicalTrials.gov or equivalent), where applicable.	Not Applicable	

Laboratory protocol	Information included in the manuscript?	In which section is the information available? (Reagents and Tools Table, Materials and Methods, Figures, Data Availability Section)
Provide DOI OR other citation details if **external detailed step-by-step protocols** are available.	Not Applicable	

Experimental study design and statistics	Information included in the manuscript?	In which section is the information available? (Reagents and Tools Table, Materials and Methods, Figures, Data Availability Section)
Include a statement about **sample size** estimate even if no statistical methods were used.	Yes	Materials and methods, Figures
Were any steps taken to minimize the effects of subjective bias when allocating animals/samples to treatment (e.g. **randomization procedure**)? If yes, have they been described?	Not Applicable	
Include a statement about **blinding** even if no blinding was done.	Not Applicable	
Describe **inclusion/exclusion criteria** if samples or animals were excluded from the analysis. Were the criteria pre-established?<br><br>If sample or data points were omitted from analysis, report if this was due to attrition or intentional exclusion and provide justification.	Not Applicable	
For every figure, are **statistical tests** justified as appropriate? Do the data meet the assumptions of the tests (e.g., normal distribution)? Describe any methods used to assess it. Is there an estimate of variation within each group of data? Is the variance similar between the groups that are being statistically compared?	Yes	Materials and methods, Figures

Sample definition and in-laboratory replication	Information included in the manuscript?	In which section is the information available? (Reagents and Tools Table, Materials and Methods, Figures, Data Availability Section)
In the figure legends: state number of times the experiment was **replicated** in laboratory.	Yes	Figures, materials and methods
In the figure legends: define whether data describe **technical or biological replicates**.	Yes	figure legends

Ethics

Ethics	Information included in the manuscript?	In which section is the information available? (Reagents and Tools Table, Materials and Methods, Figures, Data Availability Section)
Studies involving **human participants**: State details of **authority granting ethics approval** (IRB or equivalent committee(s), provide reference number for approval.	Not Applicable	
Studies involving **human participants**: Include a statement confirming that **informed consent** was obtained from all subjects and that the experiments conformed to the principles set out in the WMA Declaration of Helsinki and the Department of Health and Human Services Belmont Report.	Not Applicable	
Studies involving **human participants:** For publication of **patient photos**, include a statement confirming that consent to publish was obtained.	Not Applicable	
Studies involving experimental **animals**: State details of **authority granting ethics approva**l (IRB or equivalent committee(s), provide reference number for approval. Include a statement of compliance with ethical regulations.	Not Applicable	
Studies involving **specimen and field samples:** State if relevant **permits** obtained, provide details of authority approving study; if none were required, explain why.	Not Applicable	

Dual Use Research of Concern (DURC)	Information included in the manuscript?	In which section is the information available? (Reagents and Tools Table, Materials and Methods, Figures, Data Availability Section)
Could your study fall under dual use research restrictions? Please check biosecurity documents and list of **select agents and toxins** (CDC): https://www.selectagents.gov/sat/list.htm	Not Applicable	
If you used a select agent, is the security level of the lab appropriate and reported in the manuscript?	Not Applicable	
If a study is subject to dual use research of concern regulations, is the name of the **authority granting approval and reference number** for the regulatory approval provided in the manuscript?	Not Applicable	

Reporting

The MDAR framework recommends adoption of discipline-specific guidelines, established and endorsed through community initiatives. Journals have their own policy about requiring specific guidelines and recommendations to complement MDAR.

Adherence to community standards	Information included in the manuscript?	In which section is the information available? (Reagents and Tools Table, Materials and Methods, Figures, Data Availability Section)
State if relevant guidelines or checklists (e.g., **ICMJE, MIBBI, ARRIVE, PRISMA**) have been followed or provided.	Not Applicable	
For **tumor marker prognostic studies,** we recommend that you follow the **REMARK** reporting guidelines (see link list at top right). See author guidelines, under 'Reporting Guidelines'. Please confirm you have followed these guidelines.	Not Applicable	
For **phase II and III randomized controlled trials**, please refer to the **CONSORT** flow diagram (see link list at top right) and submit the CONSORT checklist (see link list at top right) with your submission. See author guidelines, under 'Reporting Guidelines'. Please confirm you have submitted this list.	Not Applicable	

Data Availability

Data availability	Information included in the manuscript?	In which section is the information available? (Reagents and Tools Table, Materials and Methods, Figures, Data Availability Section)
Have **primary datasets** been deposited according to the journal's guidelines (see 'Data Deposition' section) and the respective accession numbers provided in the Data Availability Section?	Yes	Data availability, some awaiting final web links
Were **human clinical and genomic datasets** deposited in a public access-controlled repository in accordance to ethical obligations to the patients and to the applicable consent agreement?	Not Applicable	
Are **computational models** that are central and integral to a study available without restrictions in a machine-readable form? Were the relevant accession numbers or links provided?	Not Applicable	
If publicly available data were reused, provide the respective **data citations in the reference list**.	Yes	Figure legends and reference list
